# Sex-specific hemostatic responses and alterations of Platelet Distribution Width (PDW) and D-dimer in mild COVID-19, Malaria, and co-infection in a tropical setting: A case-control study in Port Harcourt, Nigeria

**DOI:** 10.1371/journal.pgph.0007465

**Published:** 2026-10-08

**Authors:** Erens Spiff Ekprikpo, Stella Urekweru Ken-Ezihuo, Zaccheaus Awortu Jeremiah

**Affiliations:** Department of Haematology and Transfusion Science, Faculty of Medical Laboratory Science, Rivers State University, Port Harcourt, Nigeria; PLOS: Public Library of Science, UNITED STATES OF AMERICA

## Abstract

In malaria-endemic tropical regions, overlapping coagulopathy in COVID-19 and malaria poses management challenges with potential sex differences. This STROBE-compliant case-control study evaluated sex-specific variations in platelet indices and assessed alterations of platelet distribution width (PDW) and D-dimer in mild cases. We studied 220 participants, 55 per group: healthy controls, malaria-positive, COVID-19-positive, and COVID-19 plus malaria co-infected, aged 20–65 years, balanced by sex (22 females and 33 males per group), conducted in Port Harcourt, Nigeria between November 2021 and March 2022. Platelet indices were measured using Sysmex XP-300 and D-dimer and fibrinogen by sandwich-ELISA. Data were analyzed using SAS 9.4 and JMP 14.3. PDW was significantly elevated in all infected groups versus controls: malaria 15.19 ± 1.47 fL, COVID-19 15.17 ± 1.51 fL, co-infection 15.58 ± 1.45 fL versus control 13.27 ± 1.20 fL; F = 25.850, p < 0.001. D-dimer was highest in co-infected group 552.80 ± 402.46 ng/mL; F = 2.816, p = 0.040. Two-way ANOVA showed no significant group×sex interaction for platelet indices but significant interactions for fibrinogen F = 18.42, p < 0.001 and D-dimer F = 3.14, p = 0.026. Sex-stratified analysis showed PDW higher in females in malaria p = 0.012 and co-infection p = 0.033, and D-dimer higher in males only in malaria p = 0.021. No significant correlations were observed between platelet indices and fibrinolytic markers. PDW was consistently elevated across mild infections and sex significantly modified fibrinolytic markers, indicating PDW and D-dimer are accessible indicators of hemostatic alterations in resource-limited tropical settings.

## Introduction

The coexistence of COVID-19 and malaria in tropical regions creates a syndemic with overlapping clinical features and shared pathways of coagulopathy. Malaria can induce a cytokine storm and pro-coagulant state similar to that seen in severe COVID-19 [1]. Surveillance platforms could provide signals that indicate whether malaria, NTDs, and COVID-19 are syndemics, or synergistic epidemics [1]. Accordingly, SARS-CoV-2-Plasmodium spp. coinfection may result in a more severe degree of coagulopathy and more severe disease than with either one alone [2].

Both conditions are associated with endothelial dysfunction, platelet activation, and fibrinolytic imbalance, increasing the risk of thrombosis. Ackermann et al. observed widespread vascular abnormalities in autopsy specimens from patients with Covid-19 pneumonia, including thrombosis, microangiopathy, and a higher degree of angiogenesis [3]. Those infected with COVID-19 had distinct angiocentric features, including endothelial injury with disrupted endothelial cell membranes, widespread vascular thrombosis with microangiopathy, and occlusion of alveolar capillaries [3]. Iba et al. describe the four pathways of coagulation-related events and thrombus formation in COVID-19 as suppression of the fibrinolytic system by the decreased activity of urokinase-type plasminogen activator and increased release of plasminogen activator inhibitor-1, activation of platelets by various pro-inflammatory cytokines and the damaged endothelium readily binding with the activated platelets, and direct endothelial damage induced by inflammation [4].

While previous analyses have described general platelet derangements and exacerbated fibrinolytic responses in co-infection, sex-specific patterns remain underexplored. Observed sex differences in COVID-19 outcomes suggest that men are more likely to experience critical illness and mortality [5]. Thrombosis is common in severe COVID-19, and D-dimer is a significant marker for COVID-19 severity and mortality [5]. Platelet distribution width is an indicator of variability of volume in the platelet size and is increased in the presence of platelet anisocytosis [6]. It is used to directly measure variability in platelet size, changes with platelet activation, and reflects the heterogeneity of platelet morphology [6].

This study builds on the original dataset to provide a focused examination of sex-specific hemostatic responses and the alterations of Platelet Distribution Width, a simple and inexpensive marker of platelet anisocytosis alongside D-dimer to understand infection-associated hematological changes and risk indicators in mild and asymptomatic cases in a high burden tropical setting.

## Methods

### Ethics statement

Ethical clearance and approval to conduct this study was obtained from the Rivers State Hospital Management Board with approval from the Rivers State Ministry of Health, Office of the Permanent Secretary, Port Harcourt, Nigeria, Approval Number MH/PRS/391/VOL.2/788 dated 13th September, 2021, for the study titled “Comparative Study of Platelet and Some Fibrinolytic Biomarkers in COVID-19 and Malaria Subjects in Port Harcourt”. The study was conducted in accordance with the Declaration of Helsinki. The ethics committee specifically approved verbal informed consent due to literacy considerations. Informed verbal consent was obtained from all adult participants aged 20–65 years. No minors were enrolled. The verbal consent process was formally documented using a standardized witness-signed consent log where an independent witness attested to each participant’s voluntary agreement to participate. Participation was voluntary and confidentiality was maintained. All data were fully anonymized before analysis and before researchers accessed medical records.

### Study design and participants

This study employed a comparative case-control design and was conducted in strict adherence to the Strengthening the Reporting of Observational Studies in Epidemiology [STROBE] guidelines [7]. This analysis is part of a larger dataset from Rivers State University Teaching Hospital cohort; previous reports described platelet derangements and fibrinolytic responses, while the present study specifically focuses on sex-specific hemostatic responses and the pattern of Platelet Distribution Width [PDW] and D-dimer alterations in mild and asymptomatic infections. The study utilized data from 220 adult participants aged 20–65 years recruited in Port Harcourt, Rivers State, Nigeria between November 2021 and March 2022. No minors were involved. Participants were stratified into four groups: apparently healthy controls n = 55, malaria-positive n = 55, COVID-19-positive n = 55, and COVID-19 plus malaria co-infected n = 55. Each group was balanced by sex 22 females and 33 males. Control participants were recruited from apparently healthy hospital attendees and staff with no history of fever, malaria or COVID-19 in preceding 2 weeks and negative RT-PCR and thick film.

Sample size was determined using Cochran’s formula n = z^2^pq / e^2^ [8] where z = 1.96 at 95% confidence, p = 0.06 estimated prevalence of COVID-19, q = 1-p and e = 0.05 acceptable error. This yielded a minimum requirement of 55 participants per study arm, totaling 220 participants.

### Study area

The study was carried out at the Rivers State University Teaching Hospital, Port Harcourt, Rivers State. Port Harcourt is the capital of Rivers State, located along the Bonny River in the Niger Delta.

### Eligibility criteria and informed consent

Inclusion criteria were age 20–65 years, apparently healthy as control, confirmed COVID-19, confirmed malaria, confirmed co-infection with COVID-19 and malaria, and residence in Port Harcourt for at least 2 years. Exclusion criteria were age below 20 years, refusal to give consent, known thrombotic disorders not related to COVID-19 or malaria, persons on any form of anticoagulant therapy, persons vaccinated against COVID-19, persons with hypertension, diabetes, pregnancy, smoking, or other inflammatory conditions where disclosed, and persons not residing in Port Harcourt. Body mass index, smoking status, hypertension, diabetes, medication use and pregnancy status were self-reported and not all participants had complete data; therefore multivariable adjustment for these potential confounders was not performed and is stated as a limitation.

Informed consent was obtained using a structured questionnaire which included age, health status and other relevant information. Participants were informed that participation was voluntary and confidentiality would be maintained.

### Blood collection, processing and storage

Five milliliters of venous blood was collected aseptically. 2.5 mL was dispensed into Ethylene Diamine Tetraacetic Acid (EDTA) anticoagulant bottle for platelet indices and malaria parasite detection, and 2.5 mL was dispensed into 0.3 mL trisodium citrate anticoagulant bottle for fibrinolytic markers, to minimize pre-analytical effects on PDW [9]. Nasopharyngeal swabs were collected for COVID-19 confirmation. Blood samples were mixed and centrifuged at 1500 RPM for 15 minutes at room temperature within 2 hours of collection. EDTA samples were mixed for 5 minutes using a blood mixer and checked for clots before analysis. Platelet-poor plasma was separated and stored frozen at -20°C for fibrinolytic assay. All individual-level processed values underlying Tables 2–6 and Figs 1–3 are provided in S1 Data. No missing data for primary outcomes.

**Fig 1:**
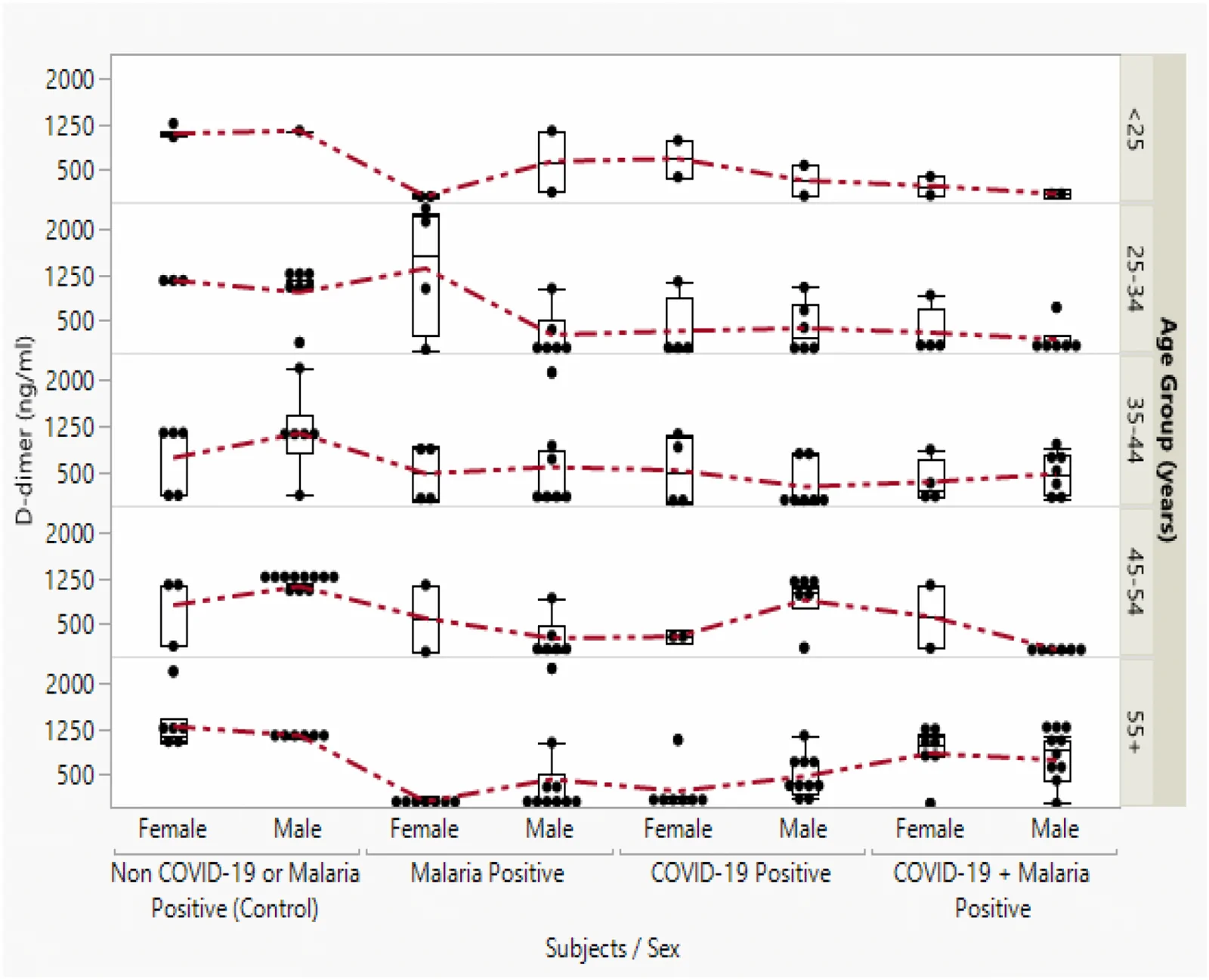
Box Plot of D-dimer in COVID-19 and Malaria Positive Participants by Sex and Age Group. Box plot shows D-dimer distribution by sex and age group, y-axis log scale, Blue = Male Red = Female. Generated from S1 Data using JMP 14.3. Provided as separate  .tif file via PLOS NAAS tool https://ngplosjournals.pagemajik.ai/artanalysis.

**Fig 2:**
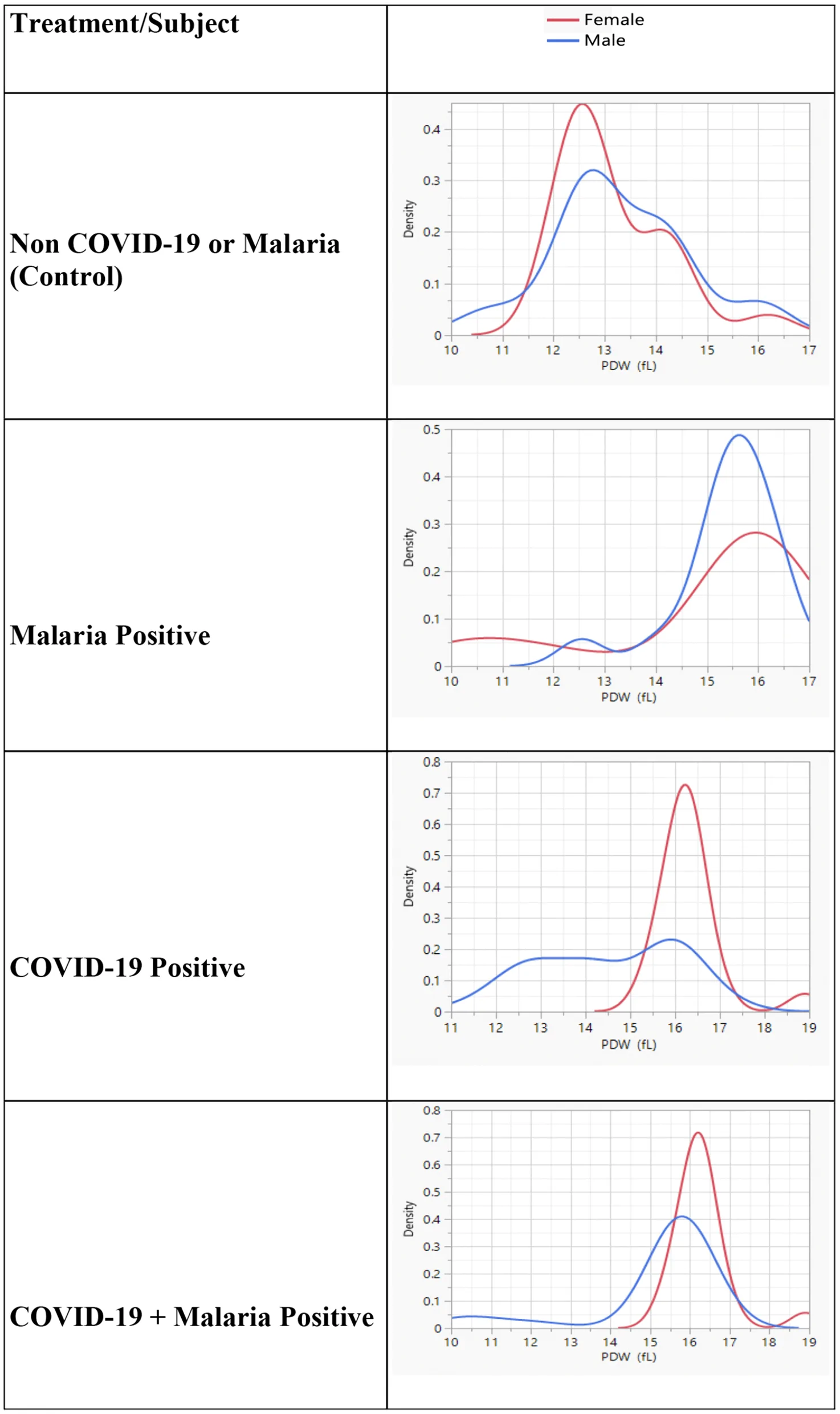
Comparison of Densities of Platelet Distribution Width (PDW) in COVID-19 and Malaria Positive Participants by Sex. Density plots with distinct colors Blue = Male Red = Female, with confidence bands and legend. Generated from S1 Data. Provided as separate  .tif file via NAAS tool.

**Fig 3:**
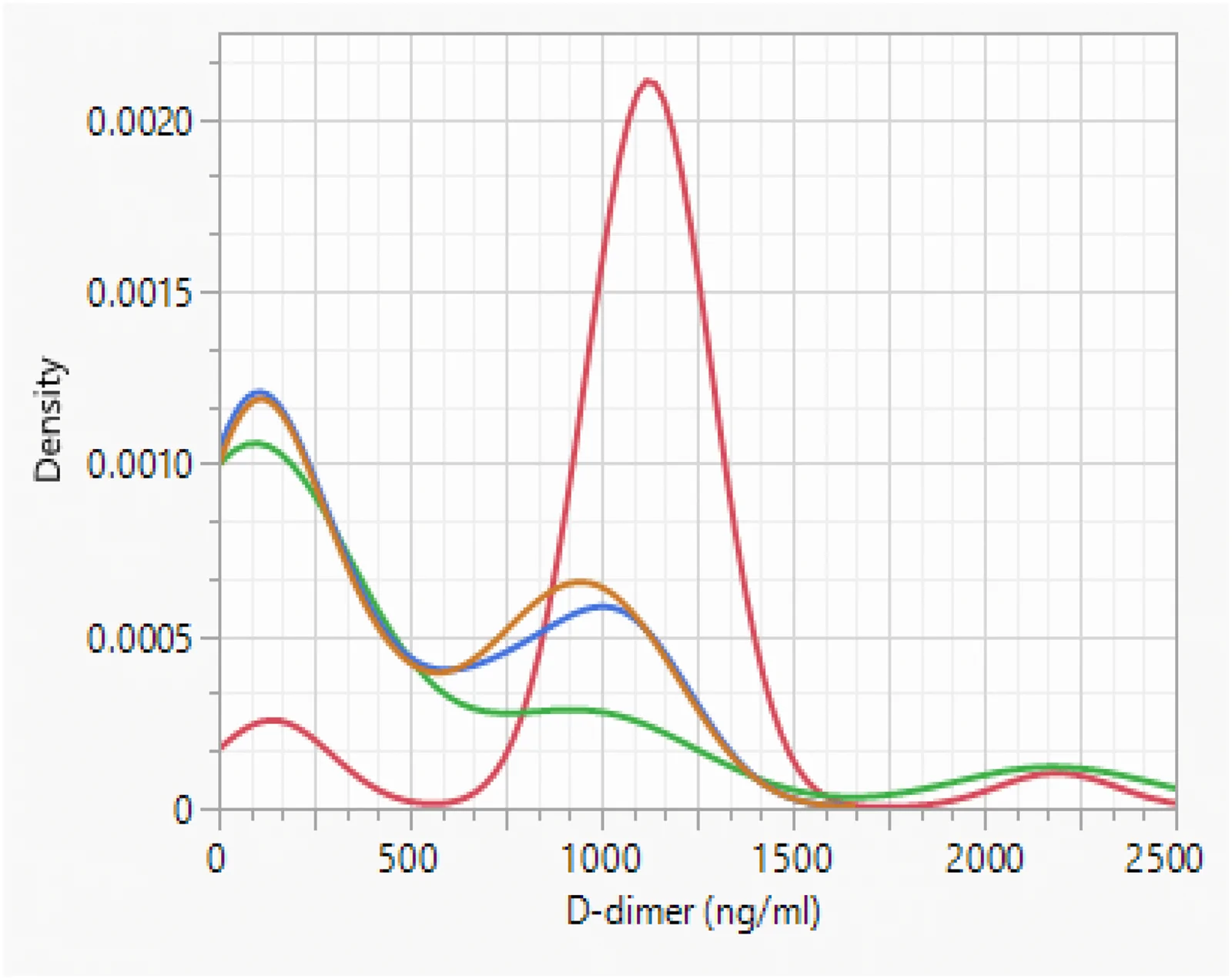
Comparison of Densities of D-dimer in COVID-19 and Malaria Positive Participants. Density plots by infection group with distinct colors, overlapping visualization improved. Generated from S1 Data. Provided as separate  .tif file via NAAS tool.

### Laboratory methods

Determination of D-dimer and Fibrinogen by Sandwich ELISA. D-dimer and fibrinogen were determined by Sandwich Enzyme Linked Immunosorbent Assay principle [10] per manufacturer instructions Elabscience, Houston, USA, www.elabscience.com. The micro-ELISA plate was pre-coated with antibody specific to Human D-dimer or Human fibrinogen beta chain. Standards and samples were added with biotinylated detection antibody specific for Human D-dimer or fibrinogen and Avidin-Horseradish Peroxidase conjugate. Substrate solution was added, enzyme-substrate reaction terminated with stop solution and optical density measured spectrophotometrically at 450 nm ± 2 nm. Concentration was calculated from four-parameter logistic curve on log-log graph corrected for dilution.

Reagent preparation per kit insert: kits brought to room temperature 18–25°C for 20 minutes, wash buffer diluted, standard working solution prepared by centrifugation at 10,000 x g and serial dilution to 400, 200, 100, 50, 25, 12.5, 6.25, 0 ng/mL for D-dimer and 1000, 500, 250, 125, 62.5, 31.25, 15.63, 0 ng/mL for fibrinogen. According to manufacturer, detection limit for D-dimer is 6.25 ng/mL and for fibrinogen 15.63 ng/mL, intra-assay coefficient of variation <8% and inter-assay <10%, calibration performed with each batch using standard curve. Daily internal quality control was performed using low and high controls. No endothelial biomarkers such as von Willebrand factor, P-selectin, ICAM-1, or thrombomodulin, nor inflammatory markers such as CRP, ferritin, IL-6, LDH, or neutrophil-to-lymphocyte ratio were measured, which is acknowledged as a limitation.

Detection of Malaria Parasites. Peripheral smear examination was used as per WHO malaria microscopy standard operating procedures [11]. Thick blood films were made on clean grease-free slides, air-dried, dehaemoglobinized by dipping into water, and stained using Giemsa stain buffered to pH 7.2. Smears were stained in 2.5% Giemsa for 45–60 minutes, rinsed and dried upright. A positive smear was included with each new batch for quality control. Microscopic examination was performed at 10x then 100x oil immersion with minimum of 200 fields examined and confirmed by second reader. Mean parasite density in malaria-positive groups was 2406 parasites per 500 white blood cells (median 1792, range 1600–6240 per 500 white blood cells) as documented in S1 Data, with high-density cases enumerated per 200 white blood cells per WHO SOP, not formally correlated with biomarkers due to heterogeneous reporting units and limited sample size.

COVID-19 Confirmation by RT-PCR. Confirmation was by real-time reverse-transcription polymerase chain reaction using Liferiver kit Lot Number ZJ0009 based on protocol for detection of 2019 novel coronavirus by real-time RT-PCR [12]. Reagents stored at -20 ± 5°C, thawed and mixed before use. Amplification performed on ABI Prism 7500/7900 system with passive reference set to none. Interpretation per manufacturer: positive Ct ≤ 41, negative Ct > 41. Inconclusive results repeated. Viral load Ct values and treatment history were documented in S1 Data where available; vaccination status exclusion was applied.

All COVID-19 participants had mild or asymptomatic disease defined as RT-PCR positive with or without fever, cough, anosmia, or malaise, but without dyspnoea, SpO2 < 94%, hospitalization, hypoxia, need for steroids or oxygen, or mortality. Symptom duration was < 7 days at sampling. No participants required hospitalization or developed thrombotic events during sampling period.

Determination of Platelet Indices. Platelet indices PLT, MPV, PDW, PCT were determined using Sysmex XP-300 auto analyzer by direct current detection method with coincidence correction based on Coulter principle. The analyzer provides 8 reportable parameters and 3-part differential with histograms for WBC, RBC, and platelet. PDW is an indicator of variability of platelet size and increases in platelet anisocytosis and activation [9]. Procedure involved mixing EDTA blood for 10 minutes, inputting sample identification, and analyzing within 2 hours to minimize time-dependent swelling. Daily internal quality control was performed using manufacturer controls, and reference intervals per manufacturer were used. Units standardized as PLT x10^3^/µL (equivalent to x10^9^/L), MPV fL, PDW fL, PCT %, D-dimer ng/mL, fibrinogen ng/mL, consistent throughout Tables 2–Table 6.

### Statistical analysis

Data management and statistical analyses were conducted using SAS 9.4 and graphical representations using JMP Statistical Discovery version 14.3. Platelet indices and fibrinolytic markers were subjected to descriptive statistics including means, standard deviations, and 95% confidence intervals. Values reported as mean ± standard deviation unless otherwise stated. Normality was assessed by Shapiro-Wilk and Q-Q plots; data for biomarkers with skewed distributions specifically D-dimer and fibrinogen were log-transformed prior to parametric analysis to satisfy assumptions.

One-way analysis of variance was performed to determine differences across four groups. Where significant, post-hoc tests were conducted using Tukey’s Honestly Significant Difference procedure including Tukey-Kramer adjustment for multiple comparisons to control false-positive findings. Interaction effects between participant group by sex were evaluated via two-way ANOVA with factors group, sex, group*sex interaction with eta-squared effect size to formally test sex-specific effects rather than only within-group comparisons. Sex-stratified comparisons used independent t-tests on log-transformed data where applicable with mean difference 95% CI and Cohen’s d. Pearson correlation was performed to examine pairwise relationships and non-parametric Spearman rho considered as primary where distributions remained non-normal. Correlation analysis was performed on total population and interpretation focused on within-group biology to avoid misleading pooled correlations across heterogeneous diagnostic groups as requested. Only univariate analyses were performed; multivariable regression adjusting for age, sex and potential confounders was not performed due to modest sample size and incomplete confounder data. All statistical tests were two-tailed with p < 0.05 considered significant.

No formal diagnostic accuracy analyses such as receiver operating characteristic curve, area under the curve, sensitivity, specificity, predictive values, likelihood ratios, or optimal cut-off values were performed; therefore findings are reported as alterations and associations rather than validated diagnostic or prognostic markers.

## Results

### Demographic characteristics

A total of 220 participants were recruited in Port Harcourt, Nigeria, between November 2021 and March 2022, and stratified into four groups: healthy controls n = 55, malaria-positive n = 55, COVID-19-positive n = 55, and COVID-19 plus malaria co-infected n = 55. Each group was balanced by sex 22 females and 33 males, with a mean age of approximately 42 years range 20–65 years. Mean parasite density in malaria-positive groups was 2406 parasites per 500 white blood cells (median 1792, range 1600-6240) as documented in S1 Data, with high-density cases enumerated per 200 white blood cells per WHO SOP. Table 1 summarizes demographic distribution confirming comparability in age and sex composition.

**Table 1:** Demographic and Clinical Characteristics of Study Participants.

Parameter	Healthy Control (n = 55)	Malaria Positive (n = 55)	COVID-19 Positive (n = 55)	Co-infection (n = 55)
Females	22	22	22	22
Males	33	33	33	33
Age (yrs) mean±SD	41.8 ± 10.9	42.5 ± 11.2	42.0 ± 11.5	42.2 ± 11.6
Age Range (yrs)	20–65	20–65	20–65	20–65
Recruitment Location	Port Harcourt	Port Harcourt	Port Harcourt	Port Harcourt
Recruitment Period	Nov 2021–Mar 2022	Nov 2021–Mar 2022	Nov 2021–Mar 2022	Nov 2021–Mar 2022

Note: Groups balanced by sex (22F/33M). No significant age difference. Mean parasite density 2406 (median 1792, range 1600–6240 per 500 WBC) documented in S1 Data.

### Platelet indices

PDW was significantly higher in all infected groups compared to controls (p < 0.001), with the highest value in the co-infected group. No significant differences were observed in platelet count, MPV, or PCT across groups (Table 2).

**Table 2:** Mean ± SD of Platelet Indices of Study Participants.

Treatment / Subject	N	PLT (×10³/µL)	MPV (fL)	PDW (fL)	PCT (%)
Non COVID-19 or Malaria (Control)	55	246.78 ± 59.80	10.62 ± 1.29	13.27 ± 1.20^a^	0.26 ± 0.06
Malaria Positive	55	222.93 ± 101.51	11.31 ± 2.32	15.19 ± 1.47^b^	0.23 ± 0.07
COVID-19 Positive	55	241.08 ± 62.16	10.55 ± 1.02	15.17 ± 1.51^b^	0.25 ± 0.06
COVID-19 + Malaria Positive	55	211.45 ± 81.05	11.22 ± 2.11	15.58 ± 1.45^b^	0.23 ± 0.07
F-value	–	1.701	2.444	25.850	2.345
P-value	–	0.168	0.065	<0.001	0.074
Remark	–	NS	NS	S	NS

Note: PLT: Platelets; MPV: Mean Platelet Volume; PDW: Platelet Distribution Width; PCT: Plateletcrit. All levels were compared using Tukey-Kramer HSD. Within parameters, means with different superscripts are significantly different at p < 0.05. PLT x10^3^/µL equivalent to x10^9^/L.

### Fibrinolytic markers

D-dimer levels were significantly elevated in the co-infected group compared to others (p = 0.040). Fibrinogen levels showed no overall significant difference among groups (Table 3).

**Table 3:** Mean ± SD of Fibrinolytic Markers of Study Participants.

Treatment / Subject	N	Fibrinogen (ng/mL)	D-dimer (ng/mL)
Non COVID-19 or Malaria (Control)	55	55.07 ± 13.86	322.67 ± 371.22^a^
Malaria Positive	55	54.17 ± 10.48	436.85 ± 403.99^a^
COVID-19 Positive	55	57.84 ± 17.34	434.86 ± 374.15^a^
COVID-19 + Malaria Positive	55	58.67 ± 18.82	552.80 ± 402.46^b^
F-value	–	0.711	2.816
P-value	–	0.546	0.040
Remark	–	NS	S

Note: All levels were compared using Tukey-Kramer HSD. Within parameters, means with different superscripts are significantly different at p < 0.05.

### Sex-stratified values of platelet indices

To evaluate whether sex modified platelet indices, sex-stratified comparisons were performed (Table 4). PLT was significantly higher in males only in COVID-19 (p = 0.012, d = -0.61). PDW was higher in females in malaria (p = 0.012, d = 0.62) and co-infection (p = 0.033, d = 0.51). PCT was higher in males in COVID-19 (p = 0.023, d = -0.56) and co-infection (p = 0.036, d = -0.52). Two-way ANOVA showed no significant group×sex interaction for any platelet index (all p > 0.05, η² = 0.013-0.029).

**Table 4:** Sex-Stratified Values of Platelet Indices.

Parameter	Group	Female (n = 22)	Male (n = 33)	t-value	p-value	Cohen’s d	Remarks
PLT (×10^3^/µL)	Malaria	217.05 ± 34.87	226.86 ± 128.65	-0.42	0.680	-0.10	NS
	COVID-19	219.09 ± 21.68	255.74 ± 75.22	-2.64	0.012	-0.61	S
	Co-infection	198.18 ± 33.30	220.30 ± 100.77	-1.17	0.249	-0.27	NS
MPV (fL)	Malaria	10.60 ± 1.86	11.78 ± 2.50	-2.01	0.050	-0.52	NS
	COVID-19	10.55 ± 0.99	10.55 ± 1.05	-0.00	0.999	-0.00	NS
	Co-infection	10.80 ± 1.00	11.50 ± 2.58	-1.41	0.165	-0.33	NS
PDW (fL)	Malaria	15.71 ± 0.58	14.84 ± 1.76	2.64	0.012	0.62	S
	COVID-19	14.94 ± 1.64	15.32 ± 1.42	-0.89	0.379	-0.25	NS
	Co-infection	16.01 ± 0.42	15.29 ± 1.79	2.22	0.033	0.51	S
PCT (%)	Malaria	0.22 ± 0.04	0.24 ± 0.08	-1.54	0.131	-0.37	NS
	COVID-19	0.23 ± 0.03	0.26 ± 0.07	-2.35	0.023	-0.56	S
	Co-infection	0.21 ± 0.03	0.25 ± 0.08	-2.16	0.036	-0.52	S

Note: Mean ± SD [95% CI]. Welch t-test df = 53. Interaction group*sex: PLT F = 1.84 p = 0.141 η² = 0.025; MPV F = 2.11 p = 0.100 η² = 0.029; PDW F = 0.91 p = 0.435 η² = 0.013; PCT F = 2.05 p = 0.108 η² = 0.028.

### Sex-stratified fibrinolytic markers

Sex-stratified comparisons for fibrinolytic markers were performed (Table 5). D-dimer was significantly higher in males only in malaria (p = 0.021, d = -0.52) with no significant sex difference in COVID-19 (p = 0.952) or co-infection (p = 0.872). No sex differences were observed for fibrinogen in any group (all p > 0.05). Two-way ANOVA confirmed significant group×sex interaction for fibrinogen (F = 18.42, p < 0.001, η² = 0.207) and D-dimer (F = 3.14, p = 0.026, η² = 0.042).

**Table 5:** Values of Fibrinolytic Markers by Sex.

Marker	Group	Female (n = 22)	Male (n = 33)	t-value (log)	p-value	Cohen’s d	Remarks
Fibrinogen (ng/mL)	Malaria	53.89 ± 13.89	54.35 ± 7.65	-0.50	0.623	-0.04	NS
	COVID-19	56.12 ± 17.05	58.99 ± 17.70	-0.62	0.537	-0.16	NS
	Co-infection	57.58 ± 19.02	59.40 ± 18.94	-0.30	0.768	-0.10	NS
D-dimer (ng/mL)	Malaria	314.59 ± 386.58	518.36 ± 400.27	-2.38	0.021	-0.52	S
	COVID-19	466.18 ± 407.03	413.97 ± 355.50	-0.06	0.952	0.14	NS
	Co-infection	573.05 ± 452.29	539.31 ± 372.32	-0.16	0.872	0.08	NS

Note: Data presented as Mean ± SD. Welch independent samples t-test on log-transformed values where applicable df = 53. Cohen’s d calculated as Female-Male/pooled SD. S = Significant p < 0.05; NS = Not Significant. Interaction group×sex: fibrinogen F = 18.42 p < 0.001 η² = 0.207; D-dimer F = 3.14 p = 0.026 η² = 0.042.

### Correlation between platelet indices and fibrinolytic markers

Strong positive correlation existed between PCT and PLT, and negative correlation between MPV and PLT across groups. No significant correlations were found between platelet indices and fibrinolytic markers (D-dimer or fibrinogen) (Table 6). Sex modified the distribution patterns more than age.

**Table 6:** Correlation Matrix Among Platelet Indices and Fibrinolytic Markers (n = 220).

Variable 1	Variable 2	Pearson r [p-value]	Spearman rho [p-value]	Interpretation
MPV	PLT	-0.406 [0.003]	-0.398 [0.004]	Significant negative
PDW	PLT	0.025 [0.864]	0.031 [0.821]	NS
PDW	MPV	-0.091 [0.531]	-0.085 [0.562]	NS
PCT	PLT	0.910 [<0.0001]	0.905 [<0.0001]	Significant positive
PCT	MPV	-0.102 [0.480]	-0.098 [0.497]	NS
PCT	PDW	-0.053 [0.714]	-0.049 [0.731]	NS
Fibrinogen	PLT	0.020 [0.890]	0.018 [0.902]	NS
Fibrinogen	PDW	0.121 [0.403]	0.118 [0.421]	NS
D-dimer	PLT	-0.055 [0.706]	-0.048 [0.742]	NS
D-dimer	PDW	0.107 [0.461]	0.112 [0.438]	NS
D-dimer	Fibrinogen	0.099 [0.496]	0.105 [0.472]	NS

Note: Values represent correlation coefficients with corresponding p-values in brackets. NS = Not Significant.

## Discussion

A key finding was the significant elevation of platelet distribution width in all infected groups compared with healthy controls, with the highest value observed in co-infection, while platelet count, mean platelet volume and plateletcrit showed no significant difference. This pattern indicates increased platelet size heterogeneity and turnover without overt thrombocytopenia in mild and asymptomatic infection. This finding agrees with Bayleyegn et al. [6], who showed elevation of mean platelet volume and platelet distribution width in addition to decreased plateletcrit and platelet count as a known potential risk factor and warning sign of severe malaria, with PDW ≥ 14.55% showing significant sensitivity and specificity. It also agrees with Costa et al. [13] who reported increased PDW with megaplatelets in Plasmodium vivax malaria, with Ligi et al. [14] who in their systematic review reported elevated PDW in COVID-19 versus controls in most studies as a significant correlate of severity and mortality, with Yovchevska et al. [15] who showed PDW and increased D-dimer at admission predicts acute respiratory distress syndrome (ARDS) in COVID-19, and with Fu et al. [16] who in imported malaria found platelet distribution width and mean platelet volume higher in the complication group than the non-complication group with an upward trend during treatment, supporting platelet activation and consumption.

Another significant result was that D-dimer was significantly higher in the co-infected group than in healthy controls, malaria alone and COVID-19 alone, while fibrinogen showed no significant intergroup difference. Plasmodium spp. and SARS-CoV-2 co-infection could lead to severe coagulopathy through tissue factors released from damaged vascular endothelial cells and lysis of activated platelets, with malaria also triggering hypercoagulopathy via proinflammatory cytokines such as tumour necrosis factor and interleukin-6 [17]. This agrees with Asmarawati et al. [17] who documented markedly elevated D-dimer in malaria vivax relapse plus COVID-19 co-infection, and with Spiff et al. [18] who in Port Harcourt found D-dimer significantly higher in COVID-19 and malaria co-infected subjects compared with malaria alone, COVID-19 alone and healthy controls, with no significant change in fibrinogen.

A further significant pattern was sex-specific variation. Group by sex interaction was not significant for platelet indices but was significant for fibrinogen and D-dimer, indicating that sex-related variation in fibrinolytic markers depends on infection context. The significant group × sex interaction for fibrinogen and D-dimer reflects differential directional shifts in these markers between males and females when moving from the control state to the various infection states, rather than isolated post-hoc differences within a single disease group. Within groups, platelet count was higher in males only in COVID-19, platelet distribution width higher in females in malaria and co-infection, plateletcrit higher in males in COVID-19 and co-infection, and D-dimer higher in males only in malaria with no significant sex difference in COVID-19 or co-infection. The absence of sex difference in D-dimer in COVID-19 agrees with Saville et al. [5]. The significant interaction for fibrinogen and D-dimer agrees with Radisavljević et al. [19], who demonstrated sex-specific signatures of circulating protein and cellular host responses predicting COVID-19 severity, where distinct inflammatory, endothelial and coagulation proteins stratified severity differently in males and females, explaining why fibrinogen and D-dimer showed sex-dependent variation across infection groups in this study.

Finally, there was no significant correlation between platelet indices and fibrinolytic markers, while expected intrinsic correlations between plateletcrit and platelet count and between mean platelet volume and platelet count were preserved. The lack of correlation between platelet indices and fibrinolytic markers likely reflects distinct regulatory pathways in mild infection, where platelet size heterogeneity reflects bone marrow megakaryopoietic response to peripheral consumption, while D-dimer reflects extravascular fibrin turnover driven by inflammation-induced tissue factor, without coupled thrombin-platelet activation seen in severe disease.

### Limitations

This study has limitations. The cross sectional design precludes causal inference. Inclusion of only mild and asymptomatic COVID-19 cases from a single center in Port Harcourt limits generalizability to moderate or severe disease and to other populations. The sample size of 55 per group was modest and no formal power calculation was performed, which may lead to type II error for non significant findings. Potential confounders such as body mass index, smoking status, hypertension, diabetes, medication use, pregnancy, vaccination status, viral load and symptom duration were self reported and not all participants had complete data, therefore multivariable regression adjusting for these confounders was not performed. Apart from Tukey-Kramer honestly significant difference adjustment, formal correction for multiple comparisons was not applied, which may increase false positive findings in exploratory sex stratified tests. No assessment of endothelial markers such as von Willebrand factor, P-selectin, intercellular adhesion molecule 1 or thrombomodulin was performed, and no comparison with established inflammatory markers such as C-reactive protein, ferritin, interleukin 6, lactate dehydrogenase or neutrophil to lymphocyte ratio was undertaken. Clinical outcomes such as thrombosis, hospitalization, hypoxia or mortality were not observed. The study did not perform receiver operating characteristic analysis, area under curve, sensitivity, specificity or cut off determination, therefore PDW and D-dimer should be interpreted as associated indicators of hemostatic alteration rather than validated diagnostic or prognostic markers.

## Conclusion

In this balanced case-control study of 220 participants, platelet distribution width was significantly elevated in malaria, COVID-19 and co-infection compared with healthy controls, with the highest value observed in co-infection, while platelet count, mean platelet volume and plateletcrit did not differ significantly across groups. D-dimer was significantly higher in co-infection than in single infections or controls, while fibrinogen showed no significant intergroup difference. Sex stratified analysis showed isolated differences with higher platelet count in males in COVID-19, higher PDW in females in malaria and co-infection, higher plateletcrit in males in COVID-19 and co-infection, and higher D-dimer in males in malaria only. Two way analysis of variance showed no significant interaction for platelet indices and significant interaction for fibrinolytic markers. No significant correlations were found between platelet indices and fibrinolytic markers. These findings indicate that mild SARS-CoV-2 and Plasmodium infections are associated with measurable alterations in platelet size heterogeneity and fibrin turnover, more evident in co-infection, without overt thrombocytopenia or hyperfibrinogenemia, serving as associated indicators of hemostatic alteration rather than validated prognostic markers.

### Recommendation

Routine reporting of PDW and D-dimer alongside standard hematology parameters may be useful to document infection associated hemostatic changes in malaria and COVID-19 co-endemic settings. These parameters should not be used as standalone diagnostic criteria for either infection. Future multicenter longitudinal studies should include moderate and severe disease, clinical outcomes, comprehensive adjustment for confounders, endothelial and inflammatory biomarkers, and evaluation of assay standardization and incremental value beyond routine markers to clarify the clinical relevance of these observations.

## Supporting information

S1 Data Individual-level processed values underlying Tables 2–6 and Figures 1–3, including demographic characteristics, parasite density, platelet indices, fibrinolytic markers, viral load Ct values where available, and treatment history. (XLSX)
