# Supplementary material for: Sex-specific hemostatic responses and alterations of Platelet Distribution Width (PDW) and D-dimer in mild COVID-19, Malaria, and co-infection in a tropical setting: A case-control study in Port Harcourt, Nigeria
Source: PLOS Glob Public Health. 2026 Oct 8;6(10):e0007465. doi: 10.1371/journal.pgph.0007465 (PMC13648994; doi:10.1371/journal.pgph.0007465)

PLOS Global Public Health

Sex-Specific Hemostatic Responses and Diagnostic Potential of Platelet Distribution Width (PDW) and D-Dimer in Mild COVID-19, Malaria, and Co-Infection in a Tropical Setting: A Case-Control Study in Port Harcourt, Nigeria.

--Manuscript Draft--

**Manuscript Number:**	PGPH-D-26-01978
**Article Type:**	Research Article
**Full Title:**	Sex-Specific Hemostatic Responses and Diagnostic Potential of Platelet Distribution Width (PDW) and D-Dimer in Mild COVID-19, Malaria, and Co-Infection in a Tropical Setting: A Case-Control Study in Port Harcourt, Nigeria.
**Short Title:**	Sex Differences in PDW and D-dimer in COVID-19–Malaria
**Corresponding Author:**	Erens Spiff Ekprikpo, PhD in Haematology and Blood Transfusion Science<br>Rivers State University<br>Port Harcourt, Rivers NIGERIA
**Order of Authors:**	Erens Spiff Ekprikpo, PhD in Haematology and Blood Transfusion Science
	Stella Urekweru Ken-Ezihuo, Haematology and Blood Transfusion Science
	Zaccheaus Awortu Jeremiah, Professor of Haematology and Blood Transfusion
**Keywords:**	Keywords: PDW, D-dimer, COVID-19, Malaria, Co-infection, Sex differences, Haemostatic response, Nigeria.
**Abstract:**	Background:<br>In malaria-endemic tropical regions, overlapping coagulopathy in COVID-19 and malaria presents diagnostic and prognostic challenges, with potential sex-specific differences. This study evaluated sex-specific variations in platelet indices and fibrinolytic markers and assessed the diagnostic utility of platelet distribution width (PDW) and D-dimer in mild or asymptomatic infections.<br>Methods:<br>A case-control study was conducted among 220 adults aged 20–65 years in Port Harcourt, Nigeria, comprising four groups (n = 55 each): healthy controls, malaria-positive, COVID-19-positive, and COVID-19/malaria co-infected individuals. Platelet indices were measured using a Sysmex XP-300 haematology analyser, while D-dimer and fibrinogen levels were determined by enzyme-linked immunosorbent assay. Statistical analyses were performed using SAS version 9.4, including one-way ANOVA with Tukey's post hoc test, Pearson correlation, and sex-stratified comparisons. Mild or asymptomatic COVID-19 cases were defined based on the absence of severe clinical symptoms and hospitalization.<br>Results:<br>PDW was significantly elevated in malaria-positive (15.21 ± 0.22 fL), COVID-19-positive (15.21 ± 0.22 fL), and co-infected participants (15.61 ± 0.21 fL) compared with controls (13.26 ± 0.17 fL; p < 0.001). D-dimer levels were highest in the co-infected group (553.42 ± 59.74 ng/mL; p = 0.040). No significant differences were observed in platelet count, mean platelet volume, plateletcrit, or fibrinogen levels across groups. Correlation analysis showed no significant association between platelet indices and fibrinolytic markers. Male participants had significantly higher D-dimer levels across all infected groups (p < 0.05) and higher fibrinogen levels in COVID-19-positive individuals (p = 0.036).<br>Conclusions:<br>COVID-19 and malaria co-infection is associated with increased fibrinolytic activity, particularly among males. PDW and D-dimer may serve as accessible and cost-effective biomarkers for detecting early hemostatic alterations in mild infections in resource-limited tropical settings.
**Additional Information:**	
**Question**	**Response**
**Financial Disclosure**	The authors received no specific funding for this work. The study was self-funded by

Enter a financial disclosure statement that describes the sources of funding for the work included in this submission and the role the funder(s) played. This includes grants and any commercial funding of the work or authors.<br><br>This statement will be typeset if the manuscript is accepted for publication.<br><br>*Please review the submission guidelines and the instructions link below for detailed requirements and guidance.*	the authors, and no external financial support was obtained.
**Competing Interests**<br><br>On behalf of all authors, disclose any competing interests that could be perceived to bias this work.<br><br>This statement will be typeset if the manuscript is accepted for publication.<br><br>*Please review the instructions link below and PLOS Global Public Health's competing interests policy to determine what information must be disclosed at submission.*	The authors have declared that no competing interests exist.
**Data Availability**<br><br>Before publication, Authors are required to make fully available and without restriction all data underlying their findings. Sharing the data underlying your findings increases the visibility, credibility, and impact of your work, while supporting transparency and future scientific discovery. Please see our PLOS Data Policy page for detailed information on this policy.	All relevant data are within the manuscript and its Supporting Information files. Additional data underlying the findings are available from the corresponding author upon reasonable request.

A **Data Availability Statement**, detailing where the data can be accessed, is required at first submission. Insert your Data Availability Statement in the box below.<br><br>Please see the data reporting section of our submission guidelines for instructions on what you need to include in your Data Availability Statement. Examples of acceptable Data Availability Statements are included here for reference:<br>1. The minimal data set [and accompanying code, where generated] is available at [repository name] via [DOI hyperlink]<br>2. No data was generated by this study. The following existing data sources were used: [dataset name] from [dataset location] available via [web address or DOI hyperlink].<br>3. For Study Protocols: No datasets were generated or analysed during the current study. All relevant data from this study will be made available upon study completion.<br><br>*PLOS allows rare exemptions to address legal and ethical concerns. If you have legal or ethical restrictions, please use the box below to detail these in full sentences for the Journal team to consider.*<br><br>This statement will be typeset if the manuscript is accepted for publication.	

Powered by Editorial Manager® and ProduXion Manager® from Aries Systems Corporation

Manuscript

Click here to access/download;Manuscript;EKPRIKPO_PLOS 2.docx

Title:

Sex-Specific Hemostatic Responses and Diagnostic Potential of Platelet Distribution Width (PDW) and D-Dimer in Mild COVID-19, Malaria, and Co-Infection in a Tropical Setting: A Case-Control Study in Port Harcourt, Nigeria.

Short Title:

Sex Differences in PDW and D-dimer in COVID-19–Malaria

Authors and Affiliations

Erens Spiff Ekprikpo[1]*, Stella Urekweru Ken-Ezihuo[1], Beauty Eruchi Echonwere-Uwikor[1], Zaccheaus Awortu Jeremiah[1]

[1]Department of Haematology and Transfusion Science, Faculty of Medical Laboratory Science, Rivers State University, Port Harcourt, Nigeria.

*Corresponding author: Erens Spiff Ekprikpo (erens.ekprikpo1@ust.edu.ng)

Abstract

Background:

In malaria-endemic tropical regions, overlapping coagulopathy in COVID-19 and malaria presents diagnostic and prognostic challenges, with potential sex-specific differences. This study evaluated sex-specific variations in platelet indices and fibrinolytic markers and assessed the

diagnostic utility of platelet distribution width (PDW) and D-dimer in mild or asymptomatic infections.

Methods:

A case-control study was conducted among 220 adults aged 20–65 years in Port Harcourt, Nigeria, comprising four groups (n = 55 each): healthy controls, malaria-positive, COVID-19-positive, and COVID-19/malaria co-infected individuals. Platelet indices were measured using a Sysmex XP-300 haematology analyser, while D-dimer and fibrinogen levels were determined by enzyme-linked immunosorbent assay. Statistical analyses were performed using SAS version 9.4, including one-way ANOVA with Tukey's post hoc test, Pearson correlation, and sex-stratified comparisons. Mild or asymptomatic COVID-19 cases were defined based on the absence of severe clinical symptoms and hospitalization.

Results:

PDW was significantly elevated in malaria-positive (15.21 ± 0.22 fL), COVID-19-positive (15.21 ± 0.22 fL), and co-infected participants (15.61 ± 0.21 fL) compared with controls (13.26 ± 0.17 fL; $p < 0.001$). D-dimer levels were highest in the co-infected group (553.42 ± 59.74 ng/mL; $p = 0.040$). No significant differences were observed in platelet count, mean platelet volume, plateletcrit, or fibrinogen levels across groups. Correlation analysis showed no significant association between platelet indices and fibrinolytic markers. Male participants had significantly higher D-dimer levels across all infected groups ($p < 0.05$) and higher fibrinogen levels in COVID-19-positive individuals ($p = 0.036$).

Conclusions:

COVID-19 and malaria co-infection is associated with increased fibrinolytic activity, particularly

among males. PDW and D-dimer may serve as accessible and cost-effective biomarkers for detecting early hemostatic alterations in mild infections in resource-limited tropical settings.

Keywords: PDW, D-dimer, COVID-19, Malaria, Co-infection, Sex differences, Haemostatic response, Nigeria

Introduction

The coexistence of COVID-19 and malaria in tropical regions creates a syndemic with overlapping clinical features and shared pathways of coagulopathy [1]**.** Both conditions are associated with endothelial dysfunction, platelet activation, and fibrinolytic imbalance, increasing the risk of thrombosis [2]**.** While previous analyses from this cohort described general platelet derangements and exacerbated fibrinolytic responses in co-infection, sex-specific patterns and the practical diagnostic value of routine haematological parameters remain underexplored [3]**.**

This study builds on the original dataset to provide a focused examination of sex-specific hemostatic responses and the potential utility of Platelet Distribution Width (PDW) a simple, inexpensive marker of platelet anisocytosis alongside D-dimer for early detection and risk stratification in mild/asymptomatic cases in a high-burden tropical setting.

Methods

Study Design and Participants

This comparative case-control study utilised data from 220 adults (55 per group) recruited in Port Harcourt, Rivers State, Nigeria. Groups included: apparently healthy controls, malaria-positive, COVID-19-positive, and COVID-19 + malaria co-infected subjects. Inclusion criteria were age 20–65 years and confirmed infection status.

Ethics Statement

Ethical approval for this study was obtained from the Rivers State Hospital Management Board Research Ethics Committee (approval number: RSHMB/RSHREC/11.21/VOL8/106). The study was conducted between November 2021 and March 2022. Participants were provided with a detailed explanation of the study purpose, procedures, potential risks, and benefits. Verbal informed consent was obtained from all participants after this explanation. The consent process was conducted in accordance with the approved protocol. All procedures were performed in accordance with the ethical standards of the institutional research committee and the Declaration of Helsinki.

Laboratory Methods

Platelet indices (PLT, MPV, PDW, PCT) were determined using the Sysmex XP-300 haematology analyser [4]**.** Fibrinolytic markers, including D-dimer and fibrinogen, were quantified using sandwich ELISA [5]**.** Malaria diagnosis was confirmed by thick blood film microscopy with Giemsa staining [6]**,** while COVID-19 status was verified by RT-PCR from nasopharyngeal swabs [7].

Statistical Analysis

Data were analysed using SAS 9.4. Descriptive statistics (mean ± SD), one-way ANOVA with Tukey's HSD post-hoc test, independent t-tests for sex comparisons, and Pearson correlation coefficients were performed. A p-value < 0.05 was considered statistically significant. Graphical representations (box plots and density curves) were generated using JMP software.

Results

Demographic characteristics of study participants

A total of 220 participants were recruited in Port Harcourt between November 2021 and March 2022 and categorized into four groups: healthy controls (n = 55), malaria-positive subjects (n = 55), COVID-19-positive subjects (n = 55), and COVID-19/malaria co-infected subjects (n = 55). Each group consisted of 22 females and 33 males. The mean age of participants was comparable across groups, ranging from 41.8 ± 10.9 to 42.5 ± 11.6 years. Participants in all groups were within the age range of 20–65 years (Table 1).

Platelet indices among study groups

The platelet indices of study participants are presented in Table 2. Platelet distribution width (PDW) differed significantly among the study groups ($F = 25.850$, $p < 0.001$). PDW values were significantly higher in malaria-positive, COVID-19-positive, and co-infected subjects compared with controls. The highest PDW value was observed among co-infected participants.

There were no statistically significant differences in platelet count (PLT), mean platelet volume (MPV), or plateletcrit (PCT) among the groups ($p > 0.05$).

Fibrinolytic markers among study groups

The fibrinolytic markers of study participants are shown in Table 3. D-dimer levels differed significantly among the study groups ($F = 2.816$, $p = 0.040$). Participants with COVID-19/malaria co-infection had the highest mean D-dimer concentration compared with the control, malaria-positive, and COVID-19-positive groups.

No statistically significant difference was observed in fibrinogen levels across the groups ($F = 0.7116$, $p = 0.5461$).

Sex-based differences in fibrinolytic markers

Sex-based comparisons of fibrinolytic markers are presented in Table 4. Among COVID-19-positive subjects, males had significantly higher fibrinogen levels than females (61.16 ± 3.73 ng/ml vs. 51.47 ± 2.48 ng/ml; $t = 2.160$, $p = 0.036$).

D-dimer levels were significantly higher in males than females among malaria-positive subjects (521.16 ± 108.89 ng/ml vs. 373.48 ± 69.34 ng/ml; $p = 0.021$), COVID-19-positive subjects (461.74 ± 67.78 ng/ml vs. 381.74 ± 96.77 ng/ml; $p = 0.013$), and co-infected subjects (530.05 ± 98.03 ng/ml vs. 518.71 ± 74.93 ng/ml; $p = 0.044$).

Distribution of D-dimer and PDW among study groups

The distribution of D-dimer levels according to sex and age group is shown in Figure 1. Higher D-dimer values were observed predominantly among male participants across the infected groups.

Figure 2 illustrates the density distribution of platelet distribution width (PDW) according to sex among the study groups. Broader PDW distributions were observed among infected participants compared with controls.

The density distribution of D-dimer levels across study groups is presented in Figure 3. Co-infected participants demonstrated greater density at higher D-dimer concentrations compared with controls and single-infection groups.

Correlation between platelet indices and fibrinolytic markers

Correlation analysis between platelet indices and fibrinolytic markers is presented in Table 5. Mean platelet volume (MPV) showed a significant negative correlation with platelet count ($r = -0.406$, $p = 0.0035$). Plateletcrit (PCT) demonstrated a strong positive correlation with platelet count ($r = 0.910$, $p < 0.0001$).

No significant correlations were observed between platelet indices and fibrinolytic markers, including fibrinogen and D-dimer ($p > 0.05$ for all comparisons).

Discussion

This study demonstrated significant alterations in fibrinolytic and platelet activation markers among individuals with COVID-19, malaria, and COVID-19/malaria co-infection in Port Harcourt. The findings highlight distinct sex-related differences in hemostatic responses and

suggest that co-infection may potentiate thrombo-inflammatory processes even in predominantly mild or asymptomatic disease presentations.

The significantly elevated D-dimer levels observed among co-infected participants indicate enhanced fibrinolytic activation associated with concurrent SARS-CoV-2 and malaria infection. This observation is consistent with previous evidence linking both infections to endothelial dysfunction, inflammatory cytokine release, and activation of coagulation pathways. COVID-19 has been associated with endothelial injury and hypercoagulability through inflammatory and immune-mediated mechanisms, whereas malaria contributes to microvascular inflammation, platelet consumption, and coagulation abnormalities. The coexistence of both infections may therefore amplify thrombo-inflammatory responses, resulting in increased D-dimer production.

Sex-specific differences were also evident in this study. Male participants demonstrated significantly higher D-dimer levels across infected groups, while males with COVID-19 additionally exhibited higher fibrinogen concentrations than females. These findings support existing reports indicating that males may experience greater inflammatory and coagulation activation during SARS-CoV-2 infection. Potential explanations include sex-based differences in innate and adaptive immune responses, hormonal regulation of inflammation, and variations in angiotensin-converting enzyme 2 (ACE2) expression. The present findings further suggest that sex may influence the hemostatic response to co-endemic infectious diseases such as COVID-19 and malaria.

Among the platelet indices evaluated, platelet distribution width (PDW) was the only parameter that differed significantly among study groups. Elevated PDW values in infected participants, particularly in co-infected subjects, may reflect increased platelet activation, heterogeneity in

platelet size, and accelerated platelet turnover during inflammatory states. Previous studies have shown that platelet activation contributes substantially to thrombo-inflammatory complications in both COVID-19 and malaria. The absence of significant differences in platelet count, mean platelet volume, and plateletcrit suggests that PDW may represent a more sensitive marker of subclinical platelet activation in mild infections.

Correlation analysis demonstrated a strong positive association between plateletcrit and platelet count, as well as a moderate inverse relationship between mean platelet volume and platelet count. However, no significant correlations were observed between platelet indices and fibrinolytic markers, indicating that platelet activation and fibrinolytic responses may occur through partially independent mechanisms during infection.

A major strength of this study is the inclusion of well-matched participant groups from a real-world tropical co-endemic setting, allowing direct comparison between malaria, COVID-19, and co-infection states. In addition, the sex-stratified analysis provided further insight into biological variability in hemostatic responses. Nevertheless, several limitations should be acknowledged. The cross-sectional design precludes causal inference and limits assessment of temporal changes in biomarker levels. Furthermore, the study predominantly included mild or asymptomatic infections, which may not fully reflect the hemostatic abnormalities observed in severe disease. The absence of longitudinal clinical outcomes also limited evaluation of the prognostic significance of the measured biomarkers.

Conclusion

COVID-19 and malaria co-infection was associated with increased fibrinolytic activation, as demonstrated by elevated D-dimer levels, while platelet distribution width emerged as a sensitive indicator of platelet activation among infected participants. Male subjects exhibited greater hemostatic activation than females, particularly with respect to D-dimer and fibrinogen levels. These findings suggest that D-dimer and PDW may serve as practical and accessible biomarkers for identifying early coagulation disturbances in malaria- and COVID-19-endemic settings.

Recommendations

1. Routine assessment of D-dimer and platelet distribution width should be considered in the evaluation of febrile patients in malaria- and COVID-19-co-endemic regions.
2. Male patients with COVID-19, malaria, or co-infection may benefit from closer monitoring for thrombo-inflammatory complications.
3. Longitudinal and multicenter studies are recommended to determine the predictive value of platelet and fibrinolytic markers for disease progression, severity, and clinical outcomes.
4. Further mechanistic studies are needed to clarify the interaction between malaria and SARS-CoV-2 infection in the activation of coagulation and inflammatory pathways.

Acknowledgments

We thank the participants and staff at Rivers State University Teaching Hospital. No specific funding was received for this study.

Data Availability Statement

All relevant data are within the manuscript and its Supporting Information files.

Funding

The authors received no specific funding for this work.

Competing Interests

The authors have declared that no competing interests exist.

References

1. Gutman JR, Lucchi NW, Cantey PT, Steinhardt LC, Samuels AM, Kamb ML, et al. Malaria and parasitic neglected tropical diseases: potential syndemics with COVID-19? Am J Trop Med Hyg. 2020;103(2):572–577.

2. Li P, Ma X, Huang G. Understanding thrombosis: the critical role of oxidative stress. Hematology. 2024;29(1):2301633.

3. Bohn MK, Higgins V, Tahmasebi H, Hall A, Liu E, Adeli K, et al. Complex biological patterns of hematology parameters in childhood necessitating age- and sex-specific reference intervals for evidence-based clinical interpretation. Int J Lab Hematol. 2020;42(6):750–760.

4. Obebinaru C, Jeremiah TA, Jeremiah ZA. Platelet indices as useful biomarkers in SARS-CoV-2 infection. Afr J Lab Haematol Transfus Sci. 2023;2(1):87–99.

5. Stang LJ. D-dimer and fibrinogen/fibrin degradation products. In: Haemostasis: Methods and Protocols. Totowa (NJ): Humana Press; 2013. p. 415–427.

6. Torres K, Bachman CM, Delahunt CB, Alarcon Baldeon J, Alava F, Gamboa Vilela D, et al. Automated microscopy for routine malaria diagnosis: a field comparison on Giemsa-stained blood films in Peru. Malar J. 2018;17(1):339.

7. Tsujimoto Y, Terada J, Kimura M, Moriya A, Motohashi A, Izumi S, et al. Diagnostic accuracy of nasopharyngeal swab, nasal swab and saliva swab samples for the detection of SARS-CoV-2 using RT-PCR. Infect Dis (Lond). 2021;53(8):581–589.

Table 1. Demographic Characteristics

Parameters	Controls (n=55)	Malaria (n=55)	COVID-19 (n=55)	Co-infection (n=55)
**Females**	22	22	22	22
**Males**	33	33	33	33
**Age (mean ± SD, yrs)**	41.8 ± 10.9	42.5 ± 11.2	42.0 ± 11.5	42.2 ± 11.6
**Age Range (yrs)**	20–65	20–65	20–65	20–65
**Recruitment Location**	Port Harcourt	Port Harcourt	Port Harcourt	Port Harcourt

Table 2. Mean ± SD of Platelet Indices of Study Subjects Infected with COVID-19 and Malaria

Treatment/Subject	N	PLT (× 10^3/µL)	MPV (fL)	PDW (fL)	PCT (%)
**Non COVID-19 or Malaria (Control)**	55	246.14± 8.73	10.63±0.18	13.26±0.17 [a]	0.26±0.01
**Malaria Positive**	55	222.46±15.07	11.35±0.34	15.21 ±0.22[b]	0.23±0.01
**COVID-19 Positive**	55	239.84±9.21	10.54±0.15	15.21 ±0.22[b]	0.25±0.01
**COVID-19+ Malaria Positive**	55	213.72±11.98	11.22±0.31	15.61 ±0.21[b]	0.23±0.01
**F-value**		1.7010	2.4444	25.850	2.345
**P-value**		0.168	0.065	<0.001	0.074
**Remark**		NS	NS	S	NS

PLT: Platelets; MPV: Mean Platelet Volume; PDW: Platelet Distribution Width; PCT: Plateletcrit. All levels were compared using Tukey-Kramer HSD. Within parameters, means with different superscripts are significantly different at $p<0.05$.

Table 3. Mean ± SD of Some Fibrinolytic Markers of Study Subjects Infected with COVID-19 and Malaria.

Treatment/Subject	N	Fibrinogen (ng/ml)	D-dimer (ng/ml)
**Non COVID-19 or Malaria (Control)**	55	55.36±2.03	319.86±51.93[a]
**Malaria Positive**	55	54.11±1.56	429.60 ±59.87[a]
**COVID-19 Positive**	55	57.48±2.57	431.34 ±55.51[a]
**COVID-19+ Malaria Positive**	55	58.32±2.79	553.42 ± 59.74[b]
**F-value**		0.7116	2.816
**P-value**		0.5461	0.040
**Remark**		NS	S

All levels were compared using Tukey-Kramer HSD. Within parameters, means with different superscripts are significantly different at $p<0.05$.

Table 4. Values of Some Fibrinolytic Markers of Study Subjects Infected with COVID-19 and Malaria by Sex

Subjects	Sex	Mean ±SD	t-value	p-value	Remarks
**Fibrinogen** (ng/ml)					
Malaria Positive	Female (n=22)	51.79±2.07	1.254	0.216	NS
	Male (n=33)	55.53±2.15			
COVID-19 Positive	Female (n=22)	51.47 ±2.48	2.160	0.036	S
	Male (n=33)	61.16 ± 3.73			
Co-infection	Female (n=22)	60.76±3.53	1.398	0.169	NS
	Male (n=33)	61.12±3.90			
**D-dimer** (ng/ml)					
Malaria Positive	Female (n=22)	373.48±69.34	1.144	0.021	S
	Male (n=33)	521.16±108.89			
COVID-19 Positive	Female (n=22)	381.74±96.77	34.947	0.013	S
	Male (n=33)	461.74±67.78			
Co-infection	Female (n=22)	518.71±74.93	0.731	0.044	S
	Male (n=33)	530.05±98.03			

All levels were compared using Tukey-Kramer HSD. Within parameters, means with different superscripts are significantly different at $p<0.05$.

Table 5. Correlation between Platelet Indices and Some Fibrinolytic Markers in the Study Groups

Variable	by Variable	Correlation	P-value	Remark
**MPV (fL)**	PLT (× 10^3/μL)	-0.406	0.0035	S
**PDW (fL)**	PLT (x 10^3/μL)	0.025	0.8640	NS
**PDW (fL)**	MPV (fL)	-0.091	0.5310	NS
**PCT (%)**	PLT (×10^3/μL)	0.910	<.0001	S
**PCT (%)**	MPV (fL)	-0.102	0.4796	NS
**PCT (%)**	PDW (fL)	-0.053	0.7140	NS
**Fibrinogen (ng/ml)**	PLT (×10^3/μL)	0.020	0.8895	NS
**Fibrinogen (ng/ml)**	MPV (fL)	-0.114	0.4297	NS
**Fibrinogen (ng/ml)**	PDW (fL)	0.121	0.4033	NS
**Fibrinogen (ng/ml)**	PCT (%)	-0.012	0.9366	NS
**D-dimer (ng/ml)**	PLT (× 10^3/μL)	-0.055	0.7060	NS
**D-dimer (ng/ml)**	MPV (fL)	-0.003	0.9815	NS
**D-dimer (ng/ml)**	PDW (fL)	0.107	0.4605	NS
**D-dimer (ng/ml)**	PCT %)	-0.064	0.6595	NS
**D-dimer (ng/ml)**	FIBRINOGEN (ng/ml)	0.099	0.4958	NS

Abbreviations: PLT: Platelets; MPV: Mean Platelet Volume; PDW: Platelet Distribution Width; PCT: Plateletcrit, Significance Levels: $p<0.01$; $p<0.0001$.

Fig 1. Box Plot of D-dimer in COVID-19 and Malaria Positive Subjects by Sex and Age Group

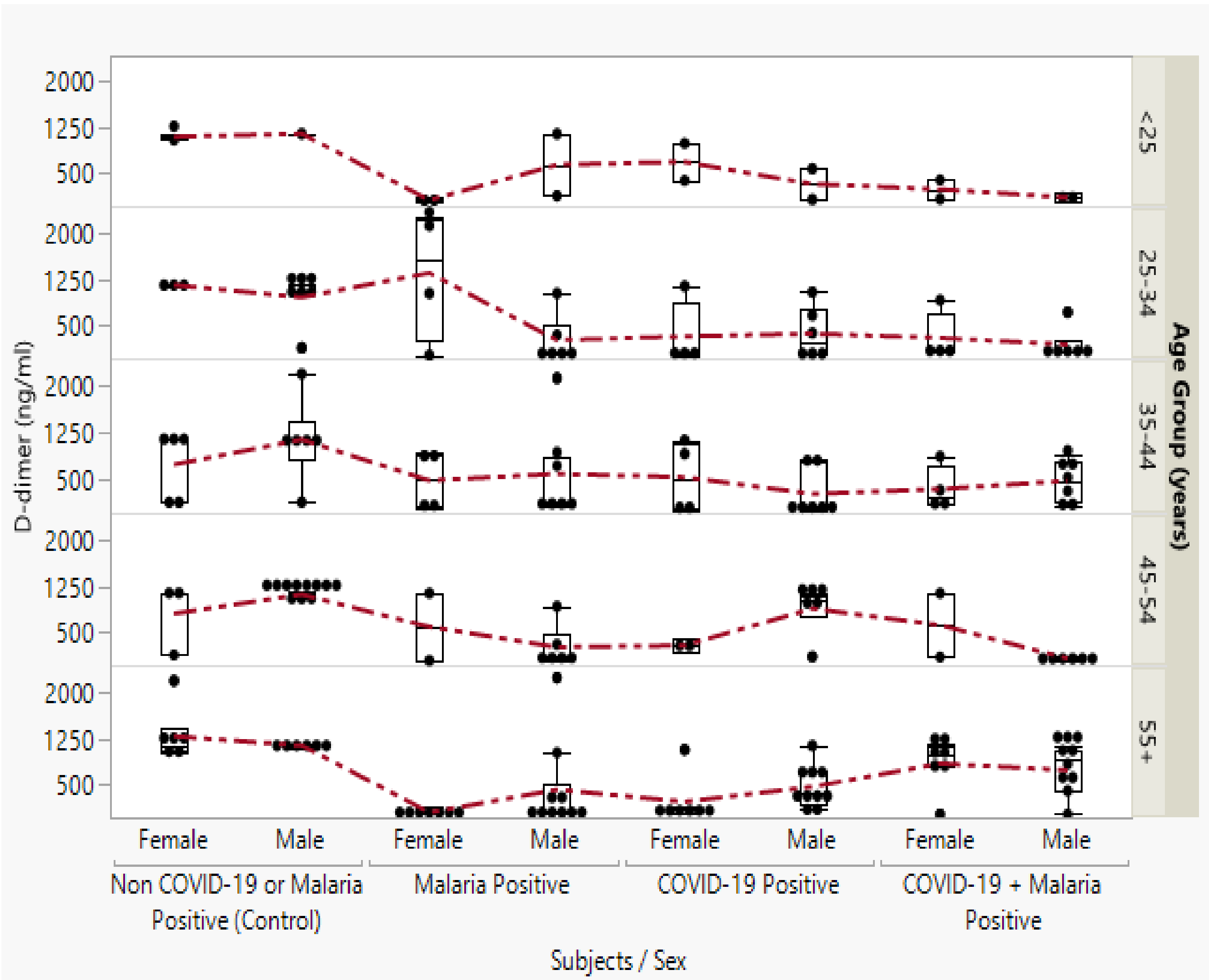

Fig 2.

**Treatment/Subject**	Female<br>Male

Comparison of Densities of Platelet Distribution Width (PDW) in COVID-19 and Malaria Positive Subjects by Sex

**Non COVID-19 or Malaria (Control)**	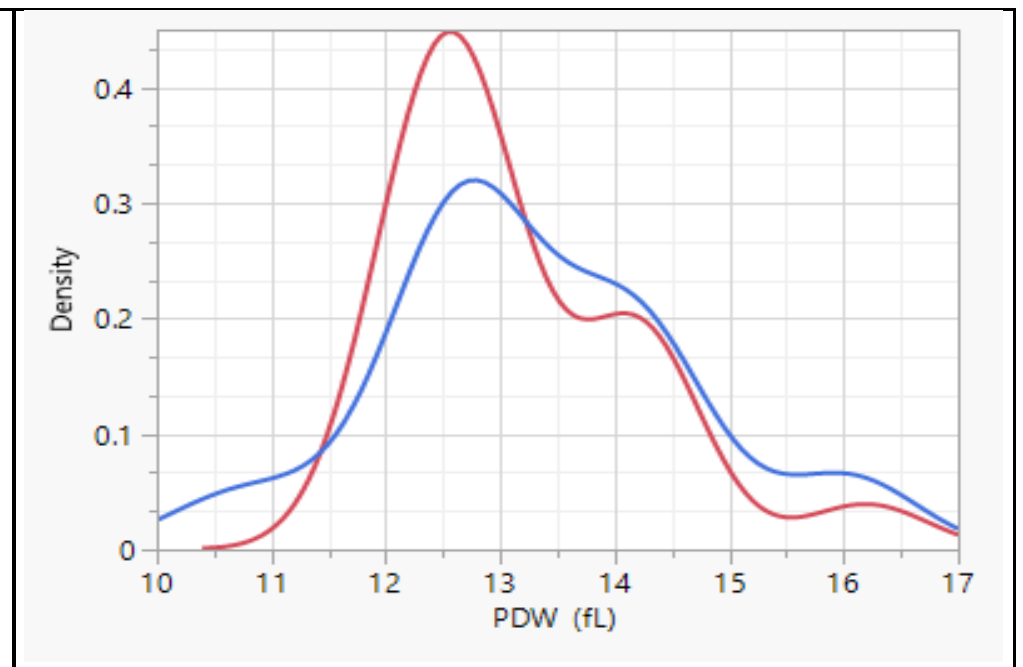
**Malaria Positive**	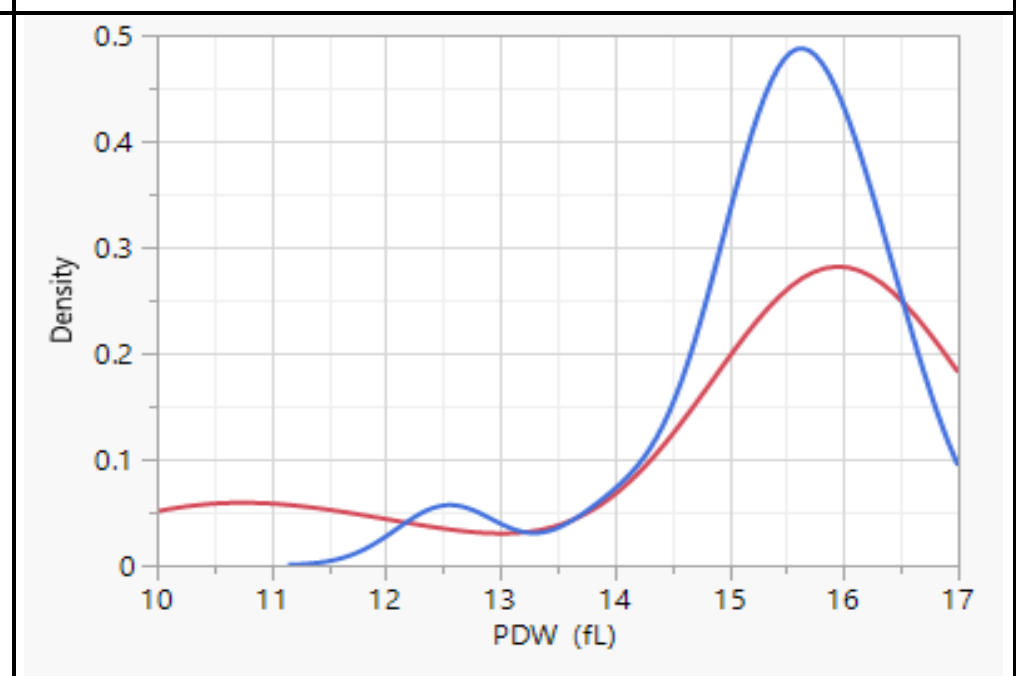
**COVID-19 Positive**	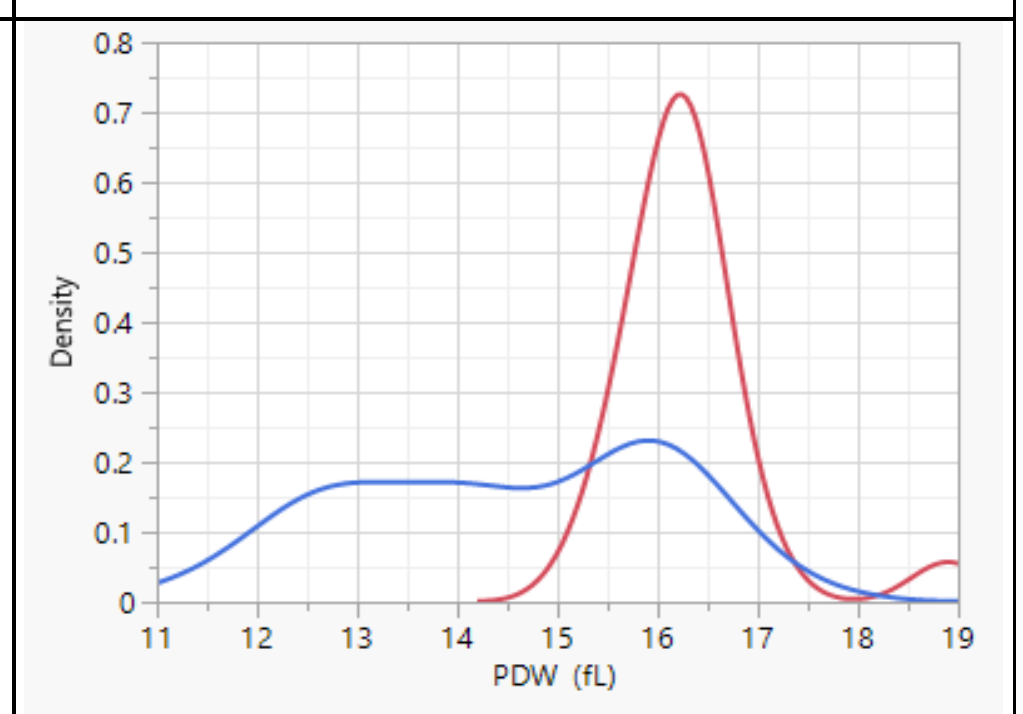
**COVID-19 + Malaria Positive**	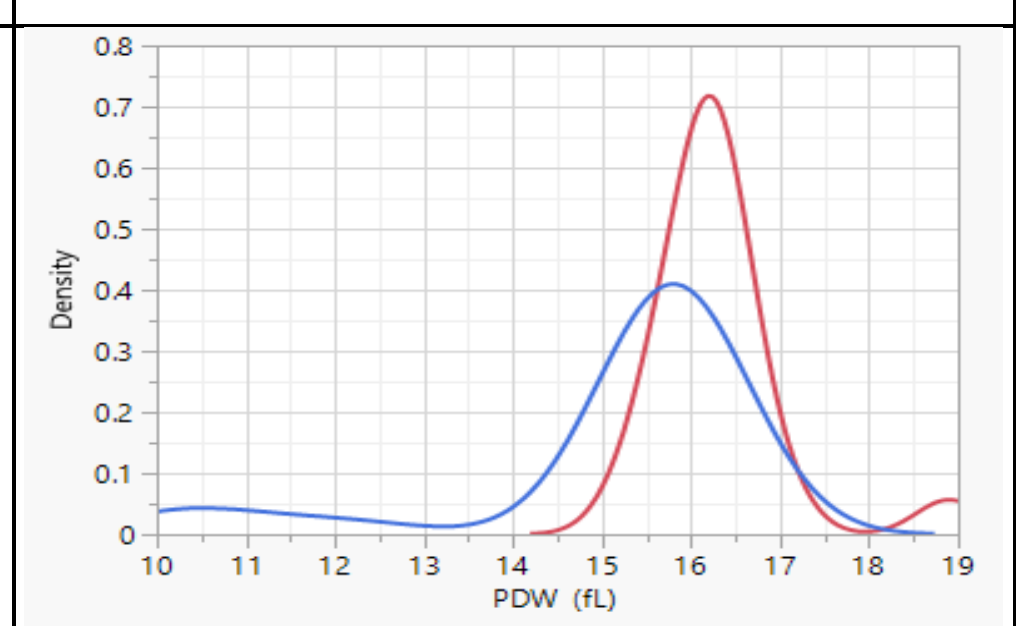

Fig 3. Comparison of Densities of D-dimer in COVID-19 and Malaria Positive Subjects

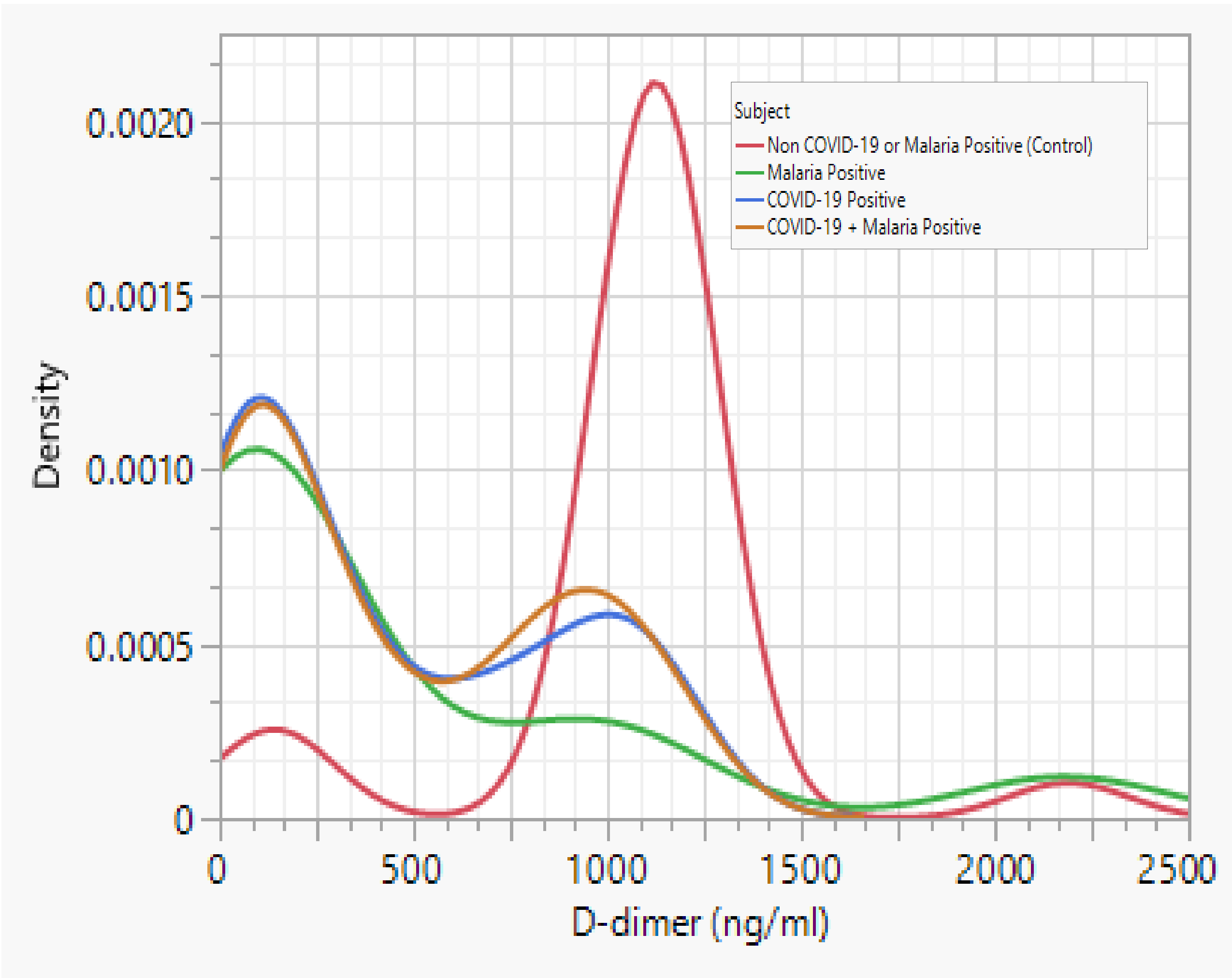

Supplement: Supplementary file 1 [file pgph.0007465.s002.pdf]
