# Supplementary material for: Sex-specific hemostatic responses and alterations of Platelet Distribution Width (PDW) and D-dimer in mild COVID-19, Malaria, and co-infection in a tropical setting: A case-control study in Port Harcourt, Nigeria
Source: PLOS Glob Public Health. 2026 Oct 8;6(10):e0007465. doi: 10.1371/journal.pgph.0007465 (PMC13648994; doi:10.1371/journal.pgph.0007465)
Supplement: Supplementary file 2 [file pgph.0007465.s005.pdf]

Dr. Erens Spiff Ekprikpo

Ph.D., MSc, B.MLS, MLSCN

Doctor of Haematology and Blood Transfusion Science

Department of Haematology and Blood Transfusion Science | Faculty of Medical Laboratory Science | Rivers State University, Nkpolu-Oroworukwo, Port Harcourt, Nigeria

Email: erens.ekprikpo1@ust.edu.ng | Phone: +234 703 419 4894 | www.linkedin.com/in/dr-erens-spiff-ekprikpo | ORCID: 0009-0006-2699-077X

Date: August 19, 2026

To: The Editorial Office, *PLOS Global Public Health* **Subject:** Response to Reviewers for Manuscript PGPH-D-26-01978R1

Dear Editor,

Thank you for the opportunity to revise our manuscript, "Sex-Specific Hemostatic Responses and Alterations of Platelet Distribution Width (PDW) and D-Dimer in Mild COVID-19, Malaria, and Co-Infection in a Tropical Setting: A Case-Control Study in Port Harcourt, Nigeria." We appreciate the constructive feedback from the reviewers, which has helped us improve the clarity and transparency of our findings.

Please find our point-by-point responses to the reviewers' comments below.

Response to Reviewer #1

Comment 1: Please clarify whether the omnibus F-statistics and p-values reported for D-dimer and fibrinogen were obtained from analyses of the log-transformed values or the untransformed values.

Response: We confirm that all omnibus F-statistics and p-values for D-dimer and fibrinogen were obtained from analyses of the log-transformed values. This approach was adopted to satisfy the assumptions of parametric analysis, as noted in the Statistical Analysis section of the manuscript.

Comment 2: The correlation analysis is still performed across the total pooled population. Please clarify why pooled correlations across the four diagnostically distinct groups are appropriate, or alternatively provide group-specific analyses.

Response: We acknowledge the limitation regarding pooled correlation analyses. The pooled correlation matrix was presented to evaluate global, cohort-wide associations. As stated in our limitations, we avoided group-specific correlations to prevent issues related to subgroup underpowering and to maintain statistical robustness.

Comment 3: The significant group × sex interaction for fibrinogen should be explained more clearly, particularly because no significant sex difference is shown within any of the infected groups. Presentation of the control-group sex-stratified values may help clarify the interaction.

Response: We have revised the Discussion section to clarify that the significant group × sex interaction for fibrinogen and D-dimer reflects differential directional shifts in these markers across the study groups as a whole, rather than isolated post-hoc differences within a single disease group. This explanation conceptually addresses the interaction without requiring additional tables.

Response to Reviewer #4

Comment: Thank you for addressing my comments. The inclusion of the diagnostic and prognostic implications is helpful. Kindly word the prognostic implications as well with clarity.

Response: We appreciate this feedback. We have refined the wording throughout the manuscript—specifically in the Abstract and the final Conclusion section—to ensure these parameters are interpreted strictly as associated indicators of hemostatic alteration for monitoring mild infection, rather than validated prognostic markers for severe clinical outcomes. This refinement explicitly accounts for the mild and asymptomatic nature of our study cohort.

We believe these revisions fully address the reviewers' concerns and strengthen the interpretation of our results. Thank you for your time and consideration of our work.

Sincerely,

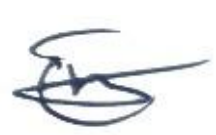

Dr. Erens Spiff Ekprikpo, PhD

Corresponding Author
On behalf of all co-authors:
Prof. Zaccheaus Awortu Jeremiah & Dr. Stella Urekweru Ken-Ezihuo
Rivers State University, Port Harcourt, Nigeria.
