# Supplementary material for: Sex-specific hemostatic responses and alterations of Platelet Distribution Width (PDW) and D-dimer in mild COVID-19, Malaria, and co-infection in a tropical setting: A case-control study in Port Harcourt, Nigeria
Source: PLOS Glob Public Health. 2026 Oct 8;6(10):e0007465. doi: 10.1371/journal.pgph.0007465 (PMC13648994; doi:10.1371/journal.pgph.0007465)
Supplement: Supplementary file 3 [file pgph.0007465.s004.docx]

 **Dr. Erens Spiff Ekprikpo**

*Ph.D., MSc, B.MLS, MLSCN*

Doctor of Haematology and Blood Transfusion Science
Department of Haematology and Blood Transfusion Science | Faculty of Medical Laboratory Science
Rivers State University, Nkpolu-Oroworukwo, Port Harcourt, Nigeria
Email: erens.ekprikpo1@ust.edu.ng | Phone: +234 703 419 4894 | [www.linkedin.com/in/dr-erens-spiff-ekprikpo](http://www.linkedin.com/in/dr-erens-spiff-ekprikpo) | ORCID: 0009-0006-2699-077X

**23 July 2026**

Dr. Miquel Vall-llosera Camps

Senior Staff Editor

PLOS Global Public Health

## Re: Response to Reviewers – Manuscript ID PGPH-D-26-01978

Title: Sex-Specific Hemostatic Responses and Alterations of Platelet Distribution Width (PDW) and D-Dimer in Mild COVID-19, Malaria, and Co-Infection in a Tropical Setting: A Case-Control Study in Port Harcourt, Nigeria. (Revised Title - previously Diagnostic Potential)

Dear Dr. Vall-llosera Camps and Reviewers,

We thank you for the constructive and detailed reviews. We have comprehensively revised the manuscript to address all journal requirements and reviewer comments. The manuscript has been re-analyzed from the raw S1 Dataset to correct inconsistencies flagged. Below is our point-by-point response. All changes are highlighted in the marked manuscript.

## JOURNAL REQUIREMENTS

### Requirement 1 – Participant Consent (Informed, type, minors, anonymization)

ADRESSED. Methods > Ethical Clearance now reads: Ethical clearance obtained from Rivers State Hospital Management Board, Rivers State Ministry of Health Approval No MH/PRS/391/VOL.2/788. The ethics committee specifically approved verbal informed consent due to literacy considerations. Informed verbal consent was obtained from all adults and documented via standardized witness-signed consent log where independent witness attested to voluntary agreement. No minors were enrolled; if minors were included, parental/guardian consent would have been obtained. All data were fully anonymized before analysis and before researchers accessed medical records. Location: Page 5, Methods.

### Requirement 2 – Financial Disclosure

ADRESSED. Updated online submission to: 'The authors received no specific funding for this work.'

### Requirement 3 – Competing Interests

ADRESSED. Updated online to: 'The authors have declared that no competing interests exist.'

### Requirement 4 – Data Availability

ADRESSED. Data Availability Statement revised to: 'All relevant data are within the manuscript and its Supporting Information files. S1 Dataset.xlsx is the minimal anonymized participant-level dataset underlying Tables 1-6 and Figures 1-3, containing de-identified group, sex, age, platelet indices, fibrinogen, D-dimer and parasite density. Figures 1-3 provided as separate .tif files via PLOS NAAS tool https://ngplosjournals.pagemajik.ai/artanalysis.' We removed 'available upon reasonable request' phrase. S1 Dataset uploaded as Supporting Information. Location: After Methods.

### Requirement 5 – Figures .tif/.eps via NAAS

ADRESSED. All figures removed from manuscript file. Figures 1-3 regenerated from S1 Dataset in JMP 14.3 and uploaded as separate .tif files via PLOS NAAS tool. Figure captions retained in manuscript. Density plots improved with distinct colors and legend Blue=Male Red=Female, Box plot y-axis log scale.

### Requirement 6 – Citation of specific works (DOI: 10.1515/cclm-2023-0625)

ADRESSED. We evaluated the suggested paper. It is relevant and has been included as Ligi et al. 2024: Platelet distribution width (PDW) as a significant correlate of COVID-19 infection severity and mortality, Clinical Chemistry and Laboratory Medicine (CCLM) 62(3):385-395. Discussed for analytical and clinical value, standardization, and implementation.

## REVIEWER #1

### Point 1 – Conclusions not supported, no ROC/AUC, diagnostic utility claims

We agree. We have substantially tempered all diagnostic claims. Title changed from 'Diagnostic Potential' to 'Alterations of PDW and D-Dimer'. Abstract, Methods, Limitations, Conclusion now explicitly state: 'No ROC/AUC analysis, sensitivity, specificity, predictive values, likelihood ratios or optimal cut-off analyses were performed; therefore PDW and D-dimer should be interpreted strictly as associated indicators of hemostatic alterations rather than validated diagnostic or prognostic markers.' No diagnostic accuracy analysis claimed.

### Point 2 – Laboratory methods too brief, STROBE not addressed, recruitment, confounders, missing data, assumptions

Expanded Methods to meet STROBE (von Elm et al. 2007). Added: Control selection – apparently healthy hospital attendees/staff with no fever, malaria or COVID-19 in preceding 2 weeks and negative RT-PCR and thick film. Sample size – Cochran formula n=z²pq/e² where z=1.96 at 95% confidence, p=0.06 prevalence Rivers State, q=1-p, e=0.05 → minimum 55 per arm total 220. Eligibility – Added exclusion of hypertension, diabetes, pregnancy, smoking, anticoagulant therapy; BMI, smoking, hypertension, diabetes, medication, pregnancy self-reported with incomplete data → stated as limitation, no multivariable adjustment. Blood collection – 2.5 mL EDTA for platelet indices/malaria, 2.5 mL citrate for fibrinolytic markers, mixed 5 min, checked for clots, processed within 2h at 1500 RPM 15 min to minimize pre-analytical effects on PDW (Budak et al. 2016), stored -20°C. ELISA – Engvall & Perlmann 1971 principle, manufacturer Elabscience Houston USA www.elabscience.com, pre-coated antibody, biotinylated detection antibody, Avidin-HRP conjugate, substrate, stop solution, OD 450±2 nm, 4-parameter logistic curve log-log, dilution factor correction, detection limits D-dimer 6.25 ng/mL fibrinogen 15.63 ng/mL, intra-assay CV <8% inter-assay <10%, calibration per batch, daily QC low/high controls. Malaria – WHO SOP WHO/HTM/GMP/MM/SOP/2016.08, Giemsa pH7.2 2.5% 45-60 min, 200 fields, second reader, positive control per batch, parasite density reported. COVID-19 – Liferiver kit Lot ZJ0009 per Corman et al. 2020, storage -20±5°C, ABI Prism 7500/7900, passive reference none, Ct ≤41 positive. Platelet indices – Sysmex XP-300 direct current detection coincidence correction Coulter principle, 8 parameters, histograms, mixed 10 min, within 2h, daily manufacturer controls, units standardized PLT x10³/µL MPV fL PDW fL PCT % D-dimer ng/mL fibrinogen ng/mL. Missing data – No missing data for primary outcomes, all processed values in S1 Dataset.

### Point 3 – Statistical analysis not rigorous, internal inconsistencies Table 4, no effect sizes, no interaction model, correlation population

We re-checked ALL results from raw S1 Dataset and corrected inconsistencies. Previously reported sex-stratified means/SDs/p-values in original submission were indeed inconsistent – thank you for catching this. CORRECTED Table 4 (Welch t-test on log-transformed where applicable): PLT malaria 217.05±34.87 vs 226.86±128.65 t=-0.42 p=0.680 d=-0.10 NS; COVID-19 219.09±21.68 vs 255.74±75.22 t=-2.64 p=0.012 d=-0.61 S; co-infection 198.18±33.30 vs 220.30±100.77 t=-1.17 p=0.249 d=-0.27 NS. MPV all NS. PDW malaria 15.71±0.58 vs 14.84±1.76 t=2.64 p=0.012 d=0.62 S; COVID-19 p=0.379 NS; co-infection 16.01±0.42 vs 15.29±1.79 t=2.22 p=0.033 d=0.51 S. PCT malaria p=0.131 NS, COVID-19 p=0.023 S, co-infection p=0.036 S. CORRECTED Table 5: Fibrinogen malaria 53.89±13.89 vs 54.35±7.65 t=-0.50 p=0.623 NS; COVID-19 p=0.537 NS; co-infection p=0.768 NS. D-dimer malaria 314.59±386.58 vs 518.36±400.27 t=-2.38 p=0.021 S; COVID-19 p=0.952 NS; co-infection p=0.872 NS. Added effect sizes Cohen's d and 95% CI, clarified values are mean±SD. Normality assessed Shapiro-Wilk and Q-Q plots, D-dimer/fibrinogen log-transformed. Added formal interaction testing: Two-way ANOVA group*sex with eta-squared: PLT F=1.84 p=0.141 η²=0.025; MPV F=2.11 p=0.100 η²=0.029; PDW F=0.91 p=0.435 η²=0.013; PCT F=2.05 p=0.108 η²=0.028 (no significant interaction for platelet indices); fibrinogen F=18.42 p<0.001 η²=0.207; D-dimer F=3.14 p=0.026 η²=0.042 (significant interaction). This formally tests sex-specific effects rather than only within-group t-tests. Correlation analysis specified as n=220 total population with Pearson r and Spearman rho, interpretation focused on within-group biology to avoid misleading pooled correlations.

### Point 4 – Data availability not sufficient

Fixed as per Journal Requirement 4. Minimal anonymized dataset S1 Dataset.xlsx uploaded.

### Point 5 – Overlap with previous publications, mild/asymptomatic definition, verbal consent documentation

Clarified: 'This analysis is part of larger dataset from Rivers State University Teaching Hospital cohort; previous reports described general platelet derangements and fibrinolytic responses (Obebinaru et al. 2023), while present study specifically focuses on sex-specific hemostatic responses and pattern of PDW and D-dimer alterations in mild/asymptomatic infections.' Mild/asymptomatic defined as RT-PCR positive with or without fever, cough, anosmia, malaise, but WITHOUT dyspnoea, SpO2<94%, hospitalization, hypoxia, need for steroids/oxygen, mortality, symptom duration <7 days, no thrombotic events during sampling. Verbal consent clarified as per Requirement 1.

### Point 6 – Language editing, terminology, units

Edited throughout: Standardized D-dimer (not D-Dimer), COVID-19/malaria co-infection (not COVID-19 + malaria), participants (not subjects), ng/mL, fL. Fixed 'PDW a simple, inexpensive marker' → 'PDW, a simple, inexpensive marker'. Table headings revised for clarity with units and footnotes.

### Point 7 – References limited (only 7)

Expanded from 7 to 19 references: Added Ackermann 2020 pulmonary vascular endothelialitis, Iba 2020 coagulopathy in COVID-19, Gutman 2020 malaria and NTDs potential syndemics, Hussein 2020 malaria and COVID-19 ties, Bayleyegn 2021 PDW ≥14.55% cut-off for malaria severity, Costa 2020 increased PDW with megaplatelets in vivax malaria, Fu 2024 PDW higher in complication group imported malaria, Ligi 2024 systematic review PDW correlate severity/mortality, Yovchevska 2022 PDW + D-dimer predicts ARDS, Asmarawati 2022 prolonged fever exaggerated hypercoagulopathy vivax relapse + COVID-19, Spiff 2023 co-infection exacerbates fibrinolytic responses, Saville 2023 sex differences D-dimer systematic review, Radisavljević 2026 sex-specific signatures circulating protein and cellular host responses, Budak 2016 pre-analytical PDW, Engvall & Perlmann 1971 ELISA principle, Corman 2020 RT-PCR, WHO 2016 microscopy SOP, von Elm 2007 STROBE, Cochran 1977 sampling.

## REVIEWER #2

### Diagnostic performance – No ROC/AUC

Same as Reviewer #1 Point 1 – tempered conclusions, title changed, no ROC/AUC claimed, reported as alterations/associations.

### Adjustment for confounding variables – BMI, smoking, hypertension, diabetes, medication, pregnancy

Groups age-matched (41.8±10.9, 42.5±11.2, 42.0±11.5, 42.2±11.6). Other confounders BMI, smoking, hypertension, diabetes, medication, pregnancy self-reported with incomplete data. Added to Methods > Eligibility and Limitations: 'Potential confounders such as BMI, smoking, hypertension, diabetes, medication use, pregnancy, vaccination status, viral load, symptom duration were self-reported and not all participants had complete data; therefore multivariable regression adjusting for these confounders was not performed and is stated as limitation. Future larger cohorts should perform multivariable regression.' This addresses that univariable analysis cannot determine independent predictors.

### Effect sizes and 95% CI – only p-values

Added Cohen's d for all sex-stratified comparisons (e.g., PLT COVID-19 d=-0.61, PDW malaria d=0.62) and eta-squared for two-way ANOVA interactions (fibrinogen η²=0.207, D-dimer η²=0.042) and mean difference 95% CI in footnotes.

### Formal interaction testing – independent t-tests insufficient

Added two-way ANOVA group*sex interaction as described in Reviewer #1 Point 3. Now Results state: no significant interaction for platelet indices but significant for fibrinogen F=18.42 p<0.001 and D-dimer F=3.14 p=0.026, indicating sex effects depend on infection context.

### Participant recruitment details and malaria parasite density

Added control recruitment details and parasite density: Mean 1850 parasites per 500 WBC (range 1600-6240 per S1 Dataset) (or mean 2406 median 1792 per Results Final Corrected file), high-density cases enumerated per 200 WBC per WHO SOP. Not formally correlated with biomarkers due to heterogeneous reporting units and limited sample size – stated in Methods and Discussion.

### ELISA details – kits, detection limits, CVs, calibration, QC

Added full details as per Reviewer #1 Point 2.

### Discussion overstated – male patients closer monitoring

Tempered. Changed from 'Males exhibit heightened fibrinolytic responses... Prioritise closer monitoring of male patients' to 'Group by sex interaction was significant for fibrinogen and D-dimer, indicating sex-related variation depends on infection context... Within groups, D-dimer higher in males only in malaria with no significant sex difference in COVID-19 or co-infection.' Recommendation now: 'Routine reporting of PDW and D-dimer may be useful to document infection-associated hemostatic changes... should not be used as standalone diagnostic criteria.'

### Correlation analysis – absence of correlations deserves biological explanation

Expanded Discussion: 'Lack of association between PDW and D-dimer or fibrinogen suggests separate regulatory pathways. PDW reflects bone marrow platelet production and peripheral consumption, while D-dimer reflects extravascular fibrin turnover driven by inflammation-induced tissue factor, without coupled thrombin-platelet activation seen in severe disease. In mild infection, compartments may be activated in parallel without direct coupling.'

### Figures 2 and 3 visualization – overlapping, colors not distinct

Regenerated Figures 1-3 from S1 Dataset in JMP 14.3. Figure 1 Box plot of D-dimer by sex and age group y-axis log scale. Figure 2 PDW density by sex and group, Figure 3 D-dimer density by infection group. Improved visualization with distinct colors (Blue=Male, Red=Female), added legend, confidence bands. Provided as separate .tif via NAAS tool.

### More comprehensive discussion – compare with other infectious diseases

Expanded Discussion to include comparison beyond COVID-19/malaria: Discussed general inflammatory responses, cited Ligi systematic review and other low-grade inflammatory conditions (Bohn et al. 2020).

### Limitations section should be expanded

Expanded from 1 sentence to 6 points covering: cross-sectional no causal inference, mild/asymptomatic only single-center limits generalizability to moderate/severe, modest sample size 55 per group no formal power calculation may lead to Type II error, no multivariable adjustment for confounders, no multiple comparisons correction apart from Tukey-Kramer HSD may increase false positives in exploratory sex-stratified tests, no endothelial markers vWF P-selectin ICAM-1 thrombomodulin, no comparison with CRP ferritin IL-6 LDH NLR, no clinical outcomes thrombosis hospitalization hypoxia mortality, no ROC/AUC.

### Discuss DOI: 10.1515/cclm-2023-0625 – laboratory biomarkers

Included as Ligi et al. 2024 and discussed for analytical and clinical value, standardization of laboratory testing, evidence-based interpretation, implementation in routine clinical practice.

### Minor editorial – terminology standardized

Standardized throughout to COVID-19-positive, malaria-positive, co-infection, consistent units PLT ×10³/µL MPV fL PDW fL PCT % D-dimer ng/mL fibrinogen ng/mL.

## REVIEWER #3

### Diagnostic utility – No ROC/AUC

Same as Reviewer #1 Point 1 – tempered, no diagnostic claims, title changed, explicit statement no ROC/AUC.

### Only univariate, need multivariable

Addressed in Reviewer #2 confounding – no multivariable due to modest sample size and incomplete confounder data, stated as limitation.

### No correction for multiple comparisons

Added in Methods and Limitations: 'Apart from Tukey-Kramer HSD adjustment, formal correction for multiple comparisons was not applied, which may increase false positive findings in exploratory sex-stratified tests.'

### No sample size justification/power

Added Cochran formula n=z²pq/e² with p=0.06 estimated prevalence Rivers State, z=1.96, e=0.05 → 55 per arm total 220. Added limitation: no formal power calculation, modest sample may lead to Type II error.

### ELISA validation, assay precision, calibration, reference intervals, QC

Added full details as per Reviewer #1 Point 2.

### Pre-analytical variables for PDW – anticoagulant, processing time, storage, analyzer QA

Added: EDTA vs citrate, mixed 5 min, checked for clots, processed within 2h to minimize pre-analytical effects on PDW (Budak et al. 2016), Sysmex XP-300 daily manufacturer controls, reference intervals per manufacturer.

### No parasite density

Added mean density and range as per Reviewer #2.

### COVID-19 characterization limited – symptom duration, viral load Ct, vaccination, previous infection, treatment

Added: symptom duration <7 days at sampling, viral load Ct values and treatment history documented in S1 Dataset where available, vaccination status exclusion applied, interpretation Ct ≤41 positive per manufacturer.

### Correlation only Pearson, no normality, need non-parametric

Added Shapiro-Wilk and Q-Q plots, log-transform for skewed D-dimer/fibrinogen, both Pearson r and Spearman rho reported as primary where non-normal.

### Endothelial dysfunction mechanisms without measuring vWF, P-selectin, ICAM-1, thrombomodulin

Moderated Discussion: Added 'This study did not measure endothelial markers such as von Willebrand factor, P-selectin, ICAM-1, or thrombomodulin, nor CRP, ferritin, IL-6, LDH, NLR. Therefore, inferences about endothelial dysfunction are indirect.'

### Sex-specific biological differences – limited mechanistic explanation

Expanded with Radisavljević et al. 2026: distinct inflammatory, endothelial and coagulation proteins stratified severity differently in males and females, explaining why fibrinogen and D-dimer showed sex-dependent variation. Also discussed hormonal and immune pathways.

### Clinical utility vs CRP, ferritin, IL-6, LDH, NLR

Added as limitation: no comparison with established inflammatory biomarkers, incremental value beyond routine markers should be evaluated in future studies.

### Cross-sectional no longitudinal follow-up, no prognostic utility

Stated as limitation, no prognostic utility claimed, conclusions tempered to 'accessible indicators of hemostatic alterations for monitoring mild infections' rather than diagnostic/prognostic tools.

### Data Availability Statement not compliant – only summary statistics

Fixed as per Journal Requirement 4 – S1 Dataset.xlsx provided.

## REVIEWER #4

Comment: The findings of hematological biomarkers in diseases with significant burden are always welcome in a public health setting. However this current manuscript lacks the detailed mention of the clinical correlation of these biomarkers. A mention of morbidity, hospitalization, hypoxia, mortality, need for steroids and other parameters, correlating with D-Dimer, PDW, Pcrit etc would greatly improve readability.

### Response to Reviewer #4

We thank Reviewer #4 for the positive comment. We have added detailed clinical correlation: Methods > COVID-19 Confirmation now states: 'All COVID-19 participants had mild or asymptomatic disease defined as RT-PCR positive with or without fever, cough, anosmia, or malaise, but WITHOUT dyspnoea, SpO2 <94%, hospitalization, hypoxia, need for steroids or oxygen, or mortality. Symptom duration was <7 days at sampling. No participants required hospitalization or developed thrombotic events during sampling period.' Discussion and Limitations now explicitly state: 'Clinical outcomes such as thrombosis, hospitalization, hypoxia, mortality, or requirement for steroids or oxygen were not assessed, as all COVID-19 participants had mild or asymptomatic disease.' Recommendation for future multicenter longitudinal studies to include moderate and severe disease, clinical outcomes, and evaluation of assay standardization and incremental value. This clarifies that our study focuses on mild infections in a resource-limited tropical setting and cannot be extrapolated to severe disease.

## SUMMARY OF MAJOR CHANGES

- Title changed from Diagnostic Potential to Alterations of PDW and D-Dimer
- Abstract corrected to match corrected Table 4 & 5: PLT higher males COVID-19 only p=0.012, PDW higher females malaria p=0.012 co-infection p=0.033, PCT higher males COVID-19 p=0.023 co-infection p=0.036, fibrinogen NO sex difference all p>0.05, D-dimer higher males only malaria p=0.021
- Methods expanded to meet STROBE and reproducibility: ELISA details, pre-analytical PDW handling, WHO malaria SOP, Liferiver kit Lot, mild definition, control selection, sample size Cochran formula, ethics witness-signed log MH/PRS/391/VOL.2/788
- Statistics re-checked from S1 Dataset: corrected Table 4 & 5 inconsistencies, added Cohen's d, eta-squared, two-way ANOVA interaction F=18.42 p<0.001 and F=3.14 p=0.026, log-transform, Shapiro-Wilk, Pearson r + Spearman rho, Tukey-Kramer HSD, no ROC/AUC
- Discussion fact-checked: Fu et al. 2024 corrected from lower to higher in complication group (Sci Rep 14:15806), Coelho 2013 → Costa 2020, Ligi 2023 → Ligi 2024 DOI 10.1515/cclm-2023-0625, Bayleyegn PDW ≥14.55%, Yovchevska ARDS, Saville no sex difference, Radisavljević 2026 sex-specific signatures
- Limitations expanded to 6 points, conclusions tempered to accessible indicators not diagnostic tools
- References expanded from 7 to 19
- Data Availability S1 Dataset.xlsx minimal anonymized dataset, Figures separate .tif via NAAS tool

We believe the revised manuscript now meets PLOS Global Public Health publication criteria: methodologically and ethically rigorous, statistically sound, data fully available, conclusions appropriately drawn and tempered.

Best Regards,

**Dr. Erens Spiff Ekprikpo, PhD, MSc, B.MLS**

Corresponding Author

Department of Haematology and Blood Transfusion Science

Faculty of Medical Laboratory Science

Rivers State University, Port Harcourt, Nigeria

Email: erens.ekprikpo1@ust.edu.ng

ORCID: 0009-0006-2699-077X

On behalf of all authors: Ekprikpo, E.S., Jeremiah, Z.A., Ken-Ezihuo, S.U.
