# Supplementary material for: Sex-specific hemostatic responses and alterations of Platelet Distribution Width (PDW) and D-dimer in mild COVID-19, Malaria, and co-infection in a tropical setting: A case-control study in Port Harcourt, Nigeria
Source: PLOS Glob Public Health. 2026 Oct 8;6(10):e0007465. doi: 10.1371/journal.pgph.0007465 (PMC13648994; doi:10.1371/journal.pgph.0007465)
Supplement: Peer review history

# Peer review: Decision Letter 0

**DOI:** 10.1371/journal.pgph.0007465.r001

**Authors:** Miquel Vall-llosera Camps

19 Jul 2026

PGPH-D-26-01978

Sex-Specific Hemostatic Responses and Diagnostic Potential of Platelet Distribution Width (PDW) and D-Dimer in Mild COVID-19, Malaria, and Co-Infection in a Tropical Setting: A Case-Control Study in Port Harcourt, Nigeria.

PLOS Global Public Health

Dear Dr. Ekprikpo,

Thank you for submitting your manuscript to PLOS Global Public Health. After careful consideration, we feel that it has merit but does not fully meet PLOS Global Public Health’s publication criteria as it currently stands. Therefore, we invite you to submit a revised version of the manuscript that addresses the points raised during the review process.

I would like to sincerely apologise for the delay you have incurred with your submission. It has been exceptionally difficult to secure reviewers to evaluate your study. We have now received four completed reviews; the comments are available below. The reviewers have raised significant scientific concerns about the study that need to be addressed in a revision.

Please revise the manuscript to address all the reviewer’s comments in a point-by-point response in order to ensure it is meeting the journal’s publication criteria. Please note that the revised manuscript will need to undergo further review, we thus cannot at this point anticipate the outcome of the evaluation process.

Please submit your revised manuscript by Sep 15 2026 11:59PM. If you will need more time than this to complete your revisions, please reply to this message or contact the journal office at globalpubhealth@plos.org. When you're ready to submit your revision, log on to https://www.editorialmanager.com/pgph/ and select the 'Submissions Needing Revision' folder to locate your manuscript file.

Please include the following items when submitting your revised manuscript:

- A letter that responds to each point raised by the editor and reviewer(s). You should upload this letter as a separate file labeled 'Response to Reviewers'.
- A marked-up copy of your manuscript that highlights changes made to the original version. You should upload this as a separate file labeled 'Revised Manuscript with Track Changes'.
- An unmarked version of your revised paper without tracked changes. You should upload this as a separate file labeled 'Manuscript'.

Guidelines for resubmitting your figure files are available below the reviewer comments at the end of this letter.

As the corresponding author, your ORCID iD is verified in the submission system and will appear in the published article. PLOS supports the use of ORCID, and we encourage all coauthors to register for an ORCID iD and use it as well. Please encourage your coauthors to verify their ORCID iD within the submission system before final acceptance, as unverified ORCID iDs will not appear in the published article. *Only*  the individual author can complete the verification step; PLOS staff *cannot*  verify ORCID iDs on behalf of authors.

We look forward to receiving your revised manuscript.

Kind regards,

Miquel Vall-llosera Camps

Senior Staff Editor

PLOS Global Public Health

Journal Requirements:

1. Please provide additional details regarding participant consent. In the ethics statement in the Methods and online submission information, please ensure that you have specified (1) whether consent was informed and (2) what type you obtained (for instance, written or verbal, and if verbal, how it was documented and witnessed). If your study included minors, state whether you obtained consent from parents or guardians. If the need for consent was waived by the ethics committee, please include this information.

If you are reporting a retrospective study of medical records or archived samples, please ensure that you have discussed whether all data were fully anonymized before you accessed them and/or whether the IRB or ethics committee waived the requirement for informed consent. If patients provided informed written consent to have data from their medical records used in research, please include this information.

2. Please amend your online Financial Disclosure statement. If you did not receive any funding for this study, please simply state: "The authors received no specific funding for this work."

3. Please update your online Competing Interests statement. If you have no competing interests to declare, please state: "The authors have declared that no competing interests exist."

4. In the online submission form, you indicated that:

"All relevant data are within the manuscript and its Supporting Information files. Additional data underlying the findings are available from the corresponding author upon reasonable request."

All PLOS journals now require all data underlying the findings described in their manuscript to be freely available to other researchers, either

a) In a public repository,

b) Within the manuscript itself, or

c) Uploaded as supplementary information.

This policy applies to all data except where public deposition would breach compliance with the protocol approved by your research ethics board. If your data cannot be made publicly available for ethical or legal reasons (e.g., public availability would compromise patient privacy), please explain your reasons by return email and your exemption request will be escalated to the editor for approval. Your exemption request will be handled independently and will not hold up the peer review process, but will need to be resolved should your manuscript be accepted for publication. One of the Editorial team will then be in touch if there are any issues.

For further assistance, you may go to: http://journals.plos.org/globalpublichealth/s/data-availability

5. Please provide separate main figure files in .tif or .eps format only and remove any figures embedded in your manuscript file. Please also ensure that all files are under our size limit of 10MB. Please leave the figure captions in the manuscript.

For more information about how to convert your figure files please see our guidelines: https://journals.plos.org/globalpublichealth/s/figures

6. If the reviewer comments include a recommendation to cite specific previously published works, please review and evaluate these publications to determine whether they are relevant and should be cited. There is no requirement to cite these works unless the editor has indicated otherwise.

[Note: HTML markup is below. Please do not edit.]

Reviewers' comments:

Reviewer’s Responses to Questions

**Comments to the Author**

1. Does this manuscript meet PLOS Global Public Health’s publication criteria?

Reviewer #1: Partly

Reviewer #2: Yes

Reviewer #3: No

Reviewer #4: Partly

**********

2. Has the statistical analysis been performed appropriately and rigorously?

Reviewer #1: No

Reviewer #2: No

Reviewer #3: No

Reviewer #4: Yes

**********

3. Have the authors made all data underlying the findings in their manuscript fully available (please refer to the Data Availability Statement at the start of the manuscript PDF file)?

The PLOS Data policy

Reviewer #1: No

Reviewer #2: Yes

Reviewer #3: No

Reviewer #4: Yes

**********

4. Is the manuscript presented in an intelligible fashion and written in standard English?

Reviewer #1: Yes

Reviewer #2: Yes

Reviewer #3: Yes

Reviewer #4: Yes

**********

Reviewer #1: The manuscript addresses a relevant question for tropical, malaria-endemic settings: whether platelet distribution width and fibrinolytic markers differ by infection status and sex among adults with mild/asymptomatic COVID-19, malaria, and co-infection. The topic is potentially useful for resource-limited clinical environments, and the balanced four-group structure is a strength. However, the manuscript in its current form requires major revision.

1. The conclusions are not fully supported by the analyses presented, particularly the claims regarding diagnostic utility. No diagnostic accuracy analyses are provided, such as ROC analysis, AUC, sensitivity, specificity, predictive values, or clinically justified cut-off values. The authors should either provide appropriate diagnostic performance analyses or substantially temper these claims.

2. The laboratory methods are described too briefly to ensure reproducibility, especially regarding ELISA procedures, sample handling, assay validation, and quality control. In addition, reporting standards for observational studies, such as STROBE, are not adequately addressed. Participant recruitment, control selection, inclusion/exclusion criteria, confounders, missing data, and statistical assumptions require clearer reporting.

3. The statistical analysis is not sufficiently rigorous as presented. Several results appear internally inconsistent and should be rechecked from the raw data. For example, some sex-stratified comparisons in Table 4 are difficult to reconcile with the reported means, standard deviations, and sample sizes. The authors should also report effect sizes and confidence intervals, clarify whether values are standard deviations or standard errors, assess normality assumptions, and consider transformation or non-parametric analyses for skewed variables such as D-dimer. Since the study emphasizes sex-specific effects, an interaction-based model would be more appropriate than separate within-group comparisons alone. The correlation analysis should also specify the population analysed and avoid potentially misleading pooled correlations across heterogeneous diagnostic groups.

4. According to journal policy, data availability is not sufficient as presented. The manuscript reports group-level means, standard deviations, p-values, and graphical summaries, but the individual-level processed values underlying these analyses and figures are not clearly provided. Unless these data are included in a Supporting Information file, availability from the corresponding author upon reasonable request does not fully satisfy the requirement to make the data underlying the findings available. The authors should provide the minimal anonymized dataset used to generate the tables, statistical analyses, and figures.

5. The manuscript should clarify whether the present analysis overlaps with previous publications from the same cohort. The authors state that previous analyses from this dataset have already described platelet derangements and fibrinolytic responses, but the extent of overlap and the specific novelty of the present manuscript are not sufficiently clear. The definition of mild/asymptomatic infection should also be described in greater clinical detail, including symptom criteria, disease duration, hospitalization status, and severity assessment. In addition, the authors should clarify the ethics process for verbal informed consent, including whether verbal consent was specifically approved and how it was documented.

6. The manuscript is generally understandable and written in acceptable English, but it requires moderate language editing to improve clarity, precision, and consistency. Terminology and units should be standardized throughout, including “D-dimer,” “COVID-19/malaria co-infection,” “participants” rather than “subjects,” and “ng/mL.” Awkward or incomplete phrasing should be corrected, such as “PDW a simple, inexpensive marker,” which should read “PDW, a simple, inexpensive marker.” Table headings and group labels should also be revised for clarity.

7. ,With only 7 entries, references are limited and should be expanded to better support claims regarding COVID-19–malaria coagulopathy, sex differences, and PDW as a platelet activation marker.

Reviewer #2: The study addresses an important public health issue by investigating sex-specific alterations in platelet indices and fibrinolytic markers among patients with COVID-19, malaria, and COVID-19/malaria co-infection in a malaria-endemic setting. The inclusion of sex-stratified analyses and the focus on readily available laboratory biomarkers such as PDW and D-dimer are strengths of the work. Nevertheless, several methodological and interpretative issues should be addressed to improve the scientific rigor and clinical relevance of the manuscript.

The authors conclude that PDW and D-dimer may serve as useful diagnostic biomarkers. However, the manuscript does not actually evaluate diagnostic performance. There are no receiver operating characteristic (ROC) analyses, area under the curve (AUC), sensitivity, specificity, predictive values, likelihood ratios, or optimal cut-off values. Without these analyses, it is difficult to support the statement regarding diagnostic utility. If diagnostic performance is one of the major objectives, these analyses should be included. Otherwise, the conclusions should be moderated to state that these biomarkers are associated with infection rather than validated diagnostic markers.

The statistical analysis would benefit from adjustment for potential confounding variables. Although the groups appear age matched, other factors that influence coagulation markers, including body mass index, smoking status, hypertension, diabetes, medication use, pregnancy status, or other inflammatory conditions, were not considered. Multivariable regression analyses would substantially strengthen the conclusions regarding the independent effects of infection status and sex on PDW and D-dimer levels.

The manuscript focuses heavily on p values but provides limited information regarding the magnitude of the observed effects. Reporting effect sizes together with 95% confidence intervals would allow readers to better appreciate the clinical significance of the observed differences rather than relying solely on statistical significance.

The study repeatedly emphasizes sex-specific differences, yet formal interaction testing between sex and disease group was not performed. Independent t tests within each group do not demonstrate that the effect of infection differs significantly between males and females. A two-way ANOVA or regression model including an interaction term between sex and infection status would provide stronger evidence supporting the conclusions regarding sex-specific responses.

It would be helpful to provide additional information regarding participant recruitment. The manuscript states that subjects had mild or asymptomatic COVID-19, but the criteria used to classify disease severity should be described in greater detail. Similarly, information regarding malaria parasite density would help readers determine whether coagulation marker levels were associated with parasite burden.

D-dimer and fibrinogen were measured using ELISA. Please provide additional methodological details regarding the commercial assay kits, manufacturer, detection limits, intra-assay and inter-assay coefficients of variation, calibration procedures, and quality control measures. These details are important for reproducibility.

The Discussion occasionally overstates the findings. For example, the manuscript suggests that male patients may benefit from closer monitoring because of greater thrombo-inflammatory activation. While this is biologically plausible, the present study did not evaluate thrombotic events or clinical outcomes. Therefore, these recommendations should be presented more cautiously.

The correlation analysis is interesting, but the absence of significant correlations between platelet indices and fibrinolytic markers deserves additional discussion. The authors should elaborate on possible biological explanations for these negative findings rather than simply reporting them. These observations may provide insight into distinct mechanisms regulating platelet activation and fibrinolysis during mild infection.

Figures 2 and 3 would benefit from improved visualization. The density plots are somewhat difficult to interpret because the groups overlap considerably and the colors are not sufficiently distinct. Adding confidence bands or presenting violin plots or boxplots alongside the density curves may improve data interpretation.

The manuscript would benefit from a more comprehensive discussion comparing the present findings with previous studies evaluating PDW and D-dimer in infectious diseases beyond COVID-19 and malaria. This would help place the results into a broader clinical context and clarify whether the observed findings are unique to this co-infection setting or reflect more general inflammatory responses.

The limitations section should be expanded. In addition to acknowledging the cross-sectional design, the authors should discuss the relatively modest sample size, the single-center nature of the study, the absence of longitudinal follow-up, the lack of adjustment for important confounders, and the limited generalizability of findings to patients with moderate or severe COVID-19.

The authors may also find it valuable to discuss the study with DOI: 10.1515/cclm-2023-0625, as it provides relevant perspectives on the interpretation and clinical application of laboratory biomarkers in clinical chemistry and laboratory medicine. Incorporating insights from this study could enrich the discussion regarding the analytical and clinical value of biomarkers such as D-dimer and platelet-related indices, particularly with respect to their implementation in routine clinical practice, standardization of laboratory testing, and evidence-based interpretation of coagulation biomarkers.

Minor editorial revisions are also recommended. There are several instances where terminology should be standardized throughout the manuscript. For example, "COVID-19-positive," "COVID-19 Positive," and "COVID-19 + malaria" are used interchangeably. Maintaining consistent terminology would improve readability. Additionally, units should be checked carefully throughout the tables, particularly for fibrinogen concentrations, to ensure they are reported using the appropriate clinical units.

Reviewer #3: I have carefully read the manuscript entitled "Sex-Specific Hemostatic Responses and Diagnostic Potential of Platelet Distribution Width (PDW) and D-Dimer in Mild COVID-19, Malaria, and Co-Infection in a Tropical Setting: A Case-Control Study in Port Harcourt, Nigeria." The study addresses an important clinical problem in malaria-endemic regions and investigates potentially useful hematological biomarkers. However, several methodological and statistical limitations substantially weaken the current conclusions. Therefore, I recommend Major Revision.

The manuscript repeatedly claims the diagnostic utility of PDW and D-dimer; however, no ROC analysis, AUC, sensitivity, specificity, predictive values, or optimal cut-off analyses have been performed. These analyses are essential before diagnostic claims can be made.

Only univariate statistical analyses were performed. Multivariable regression analyses adjusting for potential confounding variables such as age and sex are necessary to determine whether PDW and D-dimer are independent predictors.

No correction for multiple statistical comparisons has been applied despite numerous simultaneous hypothesis tests, increasing the possibility of false-positive findings.

Sample size justification and statistical power calculations are not reported, making it difficult to determine whether nonsignificant findings represent true negative results or insufficient statistical power.

The manuscript provides limited methodological details regarding ELISA validation, assay precision, calibration procedures, reference intervals, and laboratory quality control.

Important pre-analytical variables affecting platelet indices, including anticoagulant type, sample processing time, storage conditions, and analyzer quality assurance, should be described because PDW is highly sensitive to these factors.

The manuscript does not report malaria parasite density or parasitemia, preventing assessment of whether biomarker changes correlate with disease burden.

COVID-19 characterization is limited. Information regarding symptom duration, viral load (Ct values), vaccination status, previous infection, and treatment history would strengthen interpretation.

Correlation analyses rely exclusively on Pearson correlation without demonstrating data normality. Non-parametric analyses should be considered if distributions are non-normal.

The discussion proposes mechanisms involving endothelial dysfunction and thrombo-inflammatory activation without measuring endothelial biomarkers such as von Willebrand factor, P-selectin, ICAM-1, or thrombomodulin. These mechanistic interpretations should therefore be moderated.

The manuscript emphasizes sex-specific biological differences but provides limited mechanistic explanation supported by experimental evidence. Additional discussion of immune, hormonal, and coagulation pathways would improve scientific depth.

The clinical utility of PDW and D-dimer should be compared with established inflammatory biomarkers such as CRP, ferritin, IL-6, LDH, or neutrophil-to-lymphocyte ratio to better establish their incremental value.

The study is cross-sectional without longitudinal follow-up or clinical outcome assessment; therefore, conclusions regarding prognostic utility should be avoided.

The Data Availability Statement does not fully comply with PLOS policy because only summary statistics are presented. The anonymized participant-level dataset should be deposited in a public repository or provided as Supporting Information.

Overall, the manuscript addresses an important topic and has potential clinical relevance. However, substantial revisions involving statistical analyses, methodological clarification, improved data availability, and moderation of the conclusions are required before the manuscript can be considered for publication.

Reviewer #4: The findings of hematological biomarkers in diseases with significant burden are always welcome in a public health setting. however this current manuscript lacks the detailed mention of the clinical correlation of these biomarkers. A mention of morbidity, hospitalization, hypoxia, mortality, need for steroids and other parameters, correlating with D-Dimer, PDW, Pcrit etc would greatly improve readibility

**********

what does this mean?). If published, this will include your full peer review and any attached files.

**Do you want your identity to be public for this peer review?**  If you choose “no”, your identity will remain anonymous but your review may still be made public.

For information about this choice, including consent withdrawal, please see our Privacy Policy

Reviewer #1: **Yes:**  Vulcanescu Dan Dumitru

Reviewer #2: No

Reviewer #3: **Yes:**  Dr. Kovuri Umadevi

Reviewer #4: **Yes:**  Shashank Shastry

**********

[NOTE: If reviewer comments were submitted as an attachment file, they will be attached to this email and accessible via the submission site. Please log into your account, locate the manuscript record, and check for the action link "View Attachments". If this link does not appear, there are no attachment files.]

Attachment Submitted filename: Review COVID malaria.pdf

# Author comment: Author response to Decision Letter 1

**DOI:** 10.1371/journal.pgph.0007465.r002

24 Jul 2026

Attachment Submitted filename: Dr. Erens Response to Reviewers.docx.docx

# Peer review: Decision Letter 1

**DOI:** 10.1371/journal.pgph.0007465.r003

**Authors:** Sarah Jose

19 Aug 2026

PGPH-D-26-01978R1

Sex-Specific Hemostatic Responses and Alterations of Platelet Distribution Width (PDW) and D-Dimer in Mild COVID-19, Malaria, and Co-Infection in a Tropical Setting: A Case-Control Study in Port Harcourt, Nigeria.

PLOS Global Public Health

Dear Dr. Ekprikpo,

Thank you for submitting your manuscript to PLOS Global Public Health. After careful consideration, we feel that it has merit but does not fully meet PLOS Global Public Health’s publication criteria as it currently stands. Therefore, we invite you to submit a revised version of the manuscript that addresses the points raised during the review process.

The reviewers found that your revised manuscript has been much improved, with just a few clarifications required to meet the standard for publication. Please revise your manuscript to address their comments (below), providing a point-by-point response to reviewers upon resubmission.

Please submit your revised manuscript by Sep 17 2026 11:59PM. If you will need more time than this to complete your revisions, please reply to this message or contact the journal office at globalpubhealth@plos.org. When you're ready to submit your revision, log on to https://www.editorialmanager.com/pgph/ and select the 'Submissions Needing Revision' folder to locate your manuscript file.

Please include the following items when submitting your revised manuscript:

- A letter that responds to each point raised by the editor and reviewer(s). You should upload this letter as a separate file labeled 'Response to Reviewers'.
- A marked-up copy of your manuscript that highlights changes made to the original version. You should upload this as a separate file labeled 'Revised Manuscript with Track Changes'.
- An unmarked version of your revised paper without tracked changes. You should upload this as a separate file labeled 'Manuscript'.

Guidelines for resubmitting your figure files are available below the reviewer comments at the end of this letter.

As the corresponding author, your ORCID iD is verified in the submission system and will appear in the published article. PLOS supports the use of ORCID, and we encourage all coauthors to register for an ORCID iD and use it as well. Please encourage your coauthors to verify their ORCID iD within the submission system before final acceptance, as unverified ORCID iDs will not appear in the published article. *Only*  the individual author can complete the verification step; PLOS staff *cannot*  verify ORCID iDs on behalf of authors.

We look forward to receiving your revised manuscript.

Kind regards,

Sarah Jose, Ph.D.

Staff Editor

PLOS Global Public Health

Journal Requirements:

If the reviewer comments include a recommendation to cite specific previously published works, please review and evaluate these publications to determine whether they are relevant and should be cited. There is no requirement to cite these works unless the editor has indicated otherwise.

Please review your reference list to ensure that it is complete and correct. If you have cited papers that have been retracted, please include the rationale for doing so in the manuscript text, or remove these references and replace them with relevant current references. Any changes to the reference list should be mentioned in the rebuttal letter that accompanies your revised manuscript. If you need to cite a retracted article, indicate the article’s retracted status in the References list and also include a citation and full reference for the retraction notice.

Additional Editor Comments (if provided):

[Note: HTML markup is below. Please do not edit.]

Reviewers' comments:

Reviewer’s Responses to Questions

**Comments to the Author**

Reviewer #1: (No Response)

Reviewer #2: All comments have been addressed

Reviewer #4: All comments have been addressed

**********

publication criteria?

Reviewer #1: Yes

Reviewer #2: Yes

Reviewer #4: Partly

**********

3. Has the statistical analysis been performed appropriately and rigorously?

Reviewer #1: No

Reviewer #2: Yes

Reviewer #4: Yes

**********

4. Have the authors made all data underlying the findings in their manuscript fully available (please refer to the Data Availability Statement at the start of the manuscript PDF file)?

The PLOS Data policy

Reviewer #1: Yes

Reviewer #2: Yes

Reviewer #4: Yes

**********

5. Is the manuscript presented in an intelligible fashion and written in standard English?

Reviewer #1: Yes

Reviewer #2: Yes

Reviewer #4: Yes

**********

Reviewer #1: Thank you for the substantial revision. Several of the previous concerns have been adequately addressed, including the diagnostic claims, methodological detail, data availability, terminology, language, and expansion of the reference base. The revised manuscript is considerably clearer and more transparent.

The following points still require clarification:

1. Please clarify whether the omnibus F-statistics and p-values reported for D-dimer and fibrinogen were obtained from analyses of the log-transformed values or the untransformed values.

2. The correlation analysis is still performed across the total pooled population. Please clarify why pooled correlations across the four diagnostically distinct groups are appropriate, or alternatively provide group-specific analyses.

3. The significant group × sex interaction for fibrinogen should be explained more clearly, particularly because no significant sex difference is shown within any of the infected groups. Presentation of the control-group sex-stratified values may help clarify the interaction.

Reviewer #2: (No Response)

Reviewer #4: Thank you for addressing my comments. The inclusion of the diagnostic and prognostic implications is helpful. Kindly word the prognostic implications as well with clarity

**********

what does this mean?). If published, this will include your full peer review and any attached files.

**Do you want your identity to be public for this peer review?**  If you choose “no”, your identity will remain anonymous but your review may still be made public.

For information about this choice, including consent withdrawal, please see our Privacy Policy

Reviewer #1: **Yes:**  Vulcanescu Dan

Reviewer #2: No

Reviewer #4: **Yes:**  Shashank Shastry

**********

[NOTE: If reviewer comments were submitted as an attachment file, they will be attached to this email and accessible via the submission site. Please log into your account, locate the manuscript record, and check for the action link "View Attachments". If this link does not appear, there are no attachment files.]

# Author comment: Author response to Decision Letter 2

**DOI:** 10.1371/journal.pgph.0007465.r004

19 Aug 2026

Attachment Submitted filename: REsponses.pdf

# Peer review: Decision Letter 2

**DOI:** 10.1371/journal.pgph.0007465.r005

**Authors:** Julia Robinson

27 Sep 2026

Sex-Specific Hemostatic Responses and Alterations of Platelet Distribution Width (PDW) and D-Dimer in Mild COVID-19, Malaria, and Co-Infection in a Tropical Setting: A Case-Control Study in Port Harcourt, Nigeria.

PGPH-D-26-01978R2

Dear Dr Ekprikpo,

We are pleased to inform you that your manuscript 'Sex-Specific Hemostatic Responses and Alterations of Platelet Distribution Width (PDW) and D-Dimer in Mild COVID-19, Malaria, and Co-Infection in a Tropical Setting: A Case-Control Study in Port Harcourt, Nigeria.' has been provisionally accepted for publication in PLOS Global Public Health.

Before your manuscript can be formally accepted you will need to complete some formatting changes, which you will receive in a follow up email. A member of our team will be in touch with a set of requests.

Please note that your manuscript will not be scheduled for publication until you have made the required changes, so a swift response is appreciated.

IMPORTANT: The editorial review process is now complete. PLOS will only permit corrections to spelling, formatting or significant scientific errors from this point onwards. Requests for major changes, or any which affect the scientific understanding of your work, will cause delays to the publication date of your manuscript.

If your institution or institutions have a press office, please notify them about your upcoming paper to help maximize its impact. If they'll be preparing press materials, please inform our press team as soon as possible -- no later than 48 hours after receiving the formal acceptance. Your manuscript will remain under strict press embargo until 2 pm Eastern Time on the date of publication. For more information, please contact globalpubhealth@plos.org.

Thank you again for supporting Open Access publishing; we are looking forward to publishing your work in PLOS Global Public Health.

Best regards,

Julia Robinson

Executive Editor

PLOS Global Public Health

***********************************************************

Reviewer Comments (if any, and for reference):

Reviewer’s Responses to Questions

**Comments to the Author**

Reviewer #2: (No Response)

**********

publication criteria?

Reviewer #2: (No Response)

**********

3. Has the statistical analysis been performed appropriately and rigorously?

Reviewer #2: (No Response)

**********

4. Have the authors made all data underlying the findings in their manuscript fully available (please refer to the Data Availability Statement at the start of the manuscript PDF file)?

The PLOS Data policy

Reviewer #2: (No Response)

**********

5. Is the manuscript presented in an intelligible fashion and written in standard English?

Reviewer #2: (No Response)

**********

Reviewer #2: (No Response)

**********

what does this mean?). If published, this will include your full peer review and any attached files.

**Do you want your identity to be public for this peer review?**  If you choose “no”, your identity will remain anonymous but your review may still be made public.

For information about this choice, including consent withdrawal, please see our Privacy Policy

Reviewer #2: No

**********
